# CancerHubs: a systematic data mining and elaboration approach for identifying novel cancer-related protein interaction hubs

**DOI:** 10.1093/bib/bbae635

**Published:** 2024-12-07

**Authors:** Ivan Ferrari, Federica De Grossi, Giancarlo Lai, Stefania Oliveto, Giorgia Deroma, Stefano Biffo, Nicola Manfrini

**Affiliations:** INGM, Istituto Nazionale Genetica Molecolare Romeo ed Enrica Invernizzi, Milan, Italy; Department of Biosciences, University of Milan, Milan, Italy; INGM, Istituto Nazionale Genetica Molecolare Romeo ed Enrica Invernizzi, Milan, Italy; Department of Biosciences, University of Milan, Milan, Italy; INGM, Istituto Nazionale Genetica Molecolare Romeo ed Enrica Invernizzi, Milan, Italy; Department of Biosciences, University of Milan, Milan, Italy; INGM, Istituto Nazionale Genetica Molecolare Romeo ed Enrica Invernizzi, Milan, Italy; Department of Biosciences, University of Milan, Milan, Italy; INGM, Istituto Nazionale Genetica Molecolare Romeo ed Enrica Invernizzi, Milan, Italy; Department of Biosciences, University of Milan, Milan, Italy; INGM, Istituto Nazionale Genetica Molecolare Romeo ed Enrica Invernizzi, Milan, Italy; Department of Biosciences, University of Milan, Milan, Italy; INGM, Istituto Nazionale Genetica Molecolare Romeo ed Enrica Invernizzi, Milan, Italy; Department of Biosciences, University of Milan, Milan, Italy

**Keywords:** cancer, oncogene, tumour suppressor, protein hub, mutational data, clinical outcome prediction, proteomics, interactomics

## Abstract

Conventional approaches to predict protein involvement in cancer often rely on defining either aberrant mutations at the single-gene level or correlating/anti-correlating transcript levels with patient survival. These approaches are typically conducted independently and focus on one protein at a time, overlooking nucleotide substitutions outside of coding regions or mutational co-occurrences in genes within the same interaction network. Here, we present *CancerHubs*, a method that integrates unbiased mutational data, clinical outcome predictions and interactomics to define novel cancer-related protein hubs. Through this approach, we identified *TGOLN2* as a putative novel broad cancer tumour suppressor and *EFTUD2* as a putative novel multiple myeloma oncogene.

## Introduction

General methods to predict protein involvement in cancer rely on straight-forward approaches based primarily either on genomics or transcriptomics. In the first case, analyses are performed on genomic data to determine the presence of aberrant mutations in the protein coding sequence of genes. In the second case, transcript levels are analysed to define correlation or anti-correlation with overall patient survival or eventual differential abundance between cancer tissues and corresponding non-tumoural regions. A series of approaches in these directions have been elaborated and implemented and some of them are also available as user-friendly online tools. Examples include the well established PolyPhen-2 tool, which predicts possible impact of amino acid substitutions on the structure and function of human proteins [1]; CanPRedict, a combination of computational methods capable of predicting cancer-associated mutations [2]; AlloDriver, a tool to identify and analyse cancer driver genes/proteins based both on mutations and structural and dynamic protein features [3]; GEPIA [4], which is based on TCGA (https://www.cancer.gov/ccg/research/genome-sequencing/tcga) and GTEx data (https://gtexportal.org/home/) and offers expression, correlation and patient survival analyses; GSCA [5], an integrated platform for analysing genomic, pharmacogenomic, and immunogenomic cancer gene sets (https://guolab.wchscu.cn/GSCA/#/); Precog [6], a system for querying associations between genomic profiles and cancer outcomes; the Network of Cancer Genes (NCG), a database to retrieve canonical and putative cancer-driver genes based on mutational data [7] and OncoScore, a tool based on a text-mining approach to assess the specific cancer association of genes [8].

However, approaches relying solely on either mutational or clinical outcome predictions derived from single-gene data can be fallacious, as cancers are complex diseases, generally caused by the concerted dysregulation of gene sets falling into precise functional modules [9]. Indeed, emerging evidence is pointing on how altered protein–protein interactions rather than single protein modifications are the actual driving forces orchestrating tumour progression [10], suggesting to start considering oncogenes or tumour suppressors not as single entities but as components of broader cancer-related systems. Starting from this assumption, an increasing number of approaches has been proposed in order to determine the wide protein network perturbations correlated with either gene mutations [11, 12] or altered mRNA expression [13, 14] and how these can be exploited for therapy implementation [15].

Most approaches in this direction however, rely primarily on either mutational or gene expression data coupled with interactomics [16–18], neither merging the two types of information nor keeping in mind any potential correlation between gene expression and clinical outcomes.

Moreover, the vast majority of gene mutation analyses rely on the well-established assumption that only missense, non-sense and frameshift mutations affect protein functionality. Recent studies showed that also synonymous mutations can actually affect transcript stability, splicing and/or translation efficiency by altering mRNA secondary structure, splice sites or codon sequences, respectively [19–21], suggesting toreview this dogma and consider any kind of nucleotide modification as capable of affecting proper downstream protein abundance/functionality.

Here, we present *CancerHubs*, a method that combines, for the first time, unbiased mutational data, clinical outcome predictions based on gene expression and interactomic data, in order to define if, and to what extent, cancer-related proteins are part of more broad-cancer-mutated networks. By exploring the data produced, we discovered several putative novel broad cancer and cancer-specific genes and validated two of them: *TGOLN2*, a putative novel broad cancer tumour suppressor and *EFTUD2*, a new putative oncogene in Multiple Myeloma.

## Materials and methods

### The *CancerHubs* algorithm

Our study developed an R pipeline to identify novel gene hubs predicted to be highly involved in cancer (available on GitHub at https://github.com/ingmbioinfo/cancerhubs). Specifically, the *CancerHubs* framework aims to identify novel gene hubs that play a critical role in cancer by integrating mutational data, clinical outcome correlations, and interactomics. This approach identifies genes likely involved in cancer progression, by ranking them based on the newly defined ‘network score,’ a metric reflecting gene involvement/impact in a specific cancer based on the number of mutated interactors its encoded protein has.

We used several publicly available datasets to implement our method, which include:

– Mutational Data: various published sources [22–29] were used to gather mutational data across multiple cancer types. From each file, both the gene name and mutation variant columns were retained. The classification column label for each tumour was then harmonized by dividing the gene variants into ‘ORF’ and ‘NON_ORF’ mutations.
– Clinical Outcome Data: Clinical data related to patient survival was collected from the Precog Meta-Z dataset [6], which includes gene expression profiles and their correlation with survival outcomes.
– Interactomic Data: Protein–protein interaction data was retrieved from the BioGRID database [30], providing a comprehensive interactome for genes of interest.

The data were then processed and integrated in the following way:

Gene Classification: for each gene, a label was appended based on the location of mutations in its sequence: ‘ORF’, mutations falling exclusively in the ORF sequence of the gene; ‘NON_ORF’, mutations falling exclusively in non-coding regions of the gene; ‘BOTH’, mutations falling in both the ORF sequence and non-coding regions of the gene, or ‘NONE’, no mutation detected.
Precog Integration: the resulting dataframe was then integrated with Precog information, and a threshold for defining ‘cancer-relevant’ genes was introduced according to previously defined metrics [6] (Z-score = |1.96|, 95% confidence level), ensuring that the identified genes exhibited meaningful correlations with clinical outcomes while minimizing false positives [31, 32]. Each gene with a score ≥ 1.96 was labelled as ‘oncogene’, while those with a score ≤ −1.96 were labelled as ‘tumour suppressor’ [6]. All others were labelled as ‘none’. Genes absent in the mutation dataset and labelled as Precog ‘none’ were discarded, as they were unlikely to significantly affect the initiation, progression, or clinical outcome of the tumour of interest.
Filtering by Mutation Numbers: to account for biases due to differences in cohort size and mutation rates of each tumour, and considering the non-normal and skewed distribution of mutations per gene [33, 34], which is due to the intrinsic nature of mutation occurrences, the median (‘md’) and the minimum number (‘min’) of mutations across all genes per each cancer were used to filter those genes exhibiting higher mutation frequencies.
This strategy provided a form of range-based normalization allowing us to focus only on mutations with clinically relevant frequencies while minimizing noise. Specifically, ‘NON_ORF’ genes with a Precog effect were retained if their mutation frequency was ≥ ‘md + min’. For ‘NON_ORF’ genes without a Precog effect, the threshold was increased given the lack of correlation between their expression and a clinical outcome, and genes were retained only if their mutation frequency was > ‘3 ^*^ md + min’. ‘ORF’ genes, regardless of their Precog effect, were considered to be more relevant than ‘NON_ORF’ genes, and hence were retained with a lower threshold, namely if their mutation frequencies was ≥ ‘md - min’. ‘BOTH’ mutated genes were retained if their mutation frequency was ≥ ‘md - min’ in the presence of a Precog effect, or > ‘3 ^*^ md’ in the absence of a Precog effect, again because lack of correlation between gene expression and a clinical outcome requires a threshold increase. Genes without mutations but with Precog Meta-Z scores ≥2.58 or ≤ −2.58 (99% confidence level) were also retained, given their strong correlation with clinical outcomes. Genes were consequently clustered as ‘Non-Precog’ (genes that are only mutated, aka *MUT* class), ‘Precog only’ (genes that are not mutated but have a significant Meta-Z score, aka *PRECOG* class), and ‘Precog’ (genes that are mutated and also have a significant Meta-Z score, aka *MUT + PRECOG* class).
Network Score Calculation: a network score for each retained gene was calculated by multiplying the total number of protein interactors (from BioGRID) by the percentage of mutated interactors (from BioGRID), defining the number of mutated neighbors of the corresponding protein.
Output Generation: output files ranking genes based on their network scores were then generated for each tumour. Genes were subdivided into the *seed* (*PRECOG* and *MUT + PRECOG* classes) or *interactor* (*MUT* class) gene types. Additional information can be found in the Supplementary Methods section.

## Results

### Overview of the *CancerHubs* method

**Figure 1:**
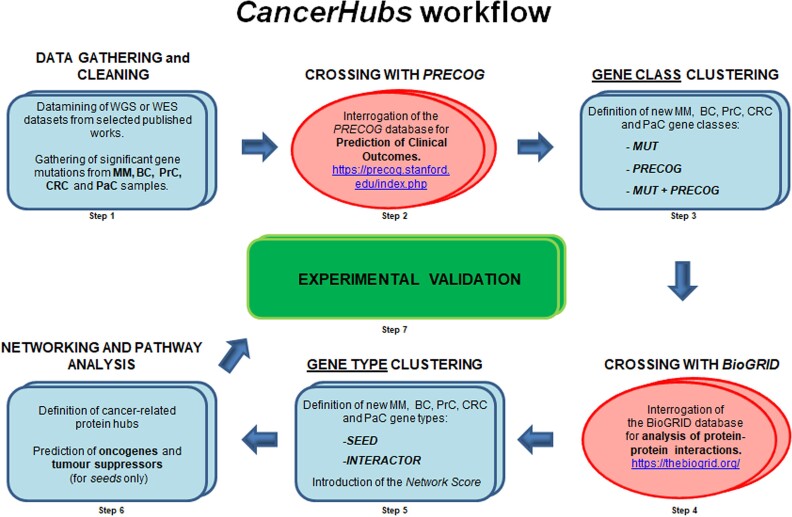
Overview of the *CancerHubs* workflow. Step 1. Mutational data was retrieved from previously published datasets and mutated gene lists were produced for each cancer selected (please refer to the Materials and Methods section for details). Step 2. Significant *Z*-scores for each gene of each cancer analysed (see Step 1) were retrieved from Precog datasets and merged/intersected with mutational data from Step 1. Step 3. Cancer-related genes were subdivided in three classes based on their mutational status and on the correlation/anti-correlation of their expression with clinical outcome predictions. We defined as *MUT* those genes that were found exclusively mutated, as *PRECOG* those genes that were not found mutated, but whose expression showed correlation/anticorrelation with a clinical outcome prediction and *MUT + PRECOG* those genes that were mutated and whose expression also correlated/anticorrelated with clinical outcome prediction. Step 4. Global protein interaction data was exported from the BioGrid database. The interactomes of each cancer-related gene (see Step 3) were defined considering as interactees only those proteins encoded by genes falling in the mutated gene lists defined in Step 1. Step 5. Cancer-related genes were now divided in two types: *seeds*, mutated genes whose expression correlated/anti-correlated with prediction of a clinical outcome and *interactors*, mutated genes whose expression did not correlate/anti-correlate with prediction of a clinical outcome. Each gene was then given a *network score*, based on the normalized number of mutant protein interactors its encoded protein had (please refer to the Materials and Methods section for details). Step 6. By ranking genes based on *network scores* we defined cancer-related protein hubs. For *seed* hubs we also give predictions regarding oncogenic or tumour suppressor behaviours. Step 7. Based on the predictions made in Step 6, we validated selected novel oncogenes/tumour suppressors proteins through wet-lab experimentation.MM: Multiple Myeloma; BC: Breast Cancer; PrC: Prostate Cancer; CRC: Colorectal Cancer; PaC: Pancreatic Cancer.

In order to define a new method to predict proteins and pathways involved in cancer disease, we implemented an approach based on merging: (i) mutational data, (ii) correlation of gene expression with clinical outcomes, and (iii) interactomics (Fig. 1 and Supplementary Fig. S1). Our final aim was to rank genes that were either mutated or whose expression correlated with a clinical outcome, based on the capacity of their encoded protein to interact with other cancer-mutated proteins. In so doing we planned to define protein hubs and networks with a potentially high impact/involvement in cancer disease.

At first, we selected five representative cancers, a liquid tumour and four types of adenocarcinomas, and, datamining the published literature, we retrieved mutational data derived from clinical samples (Step 1).

As liquid tumour we selected multiple myeloma (MM), a haematological malignancy (data retrieved from [22]), while as adenocarcinomas we analysed Breast Cancer (BC) (data retrieved from [23, 24], Prostate Cancer (PrC) (data retrieved from [25]), ColoRectal Cancer (CRC) (data retrieved from the CRC atlas [26]) and ductal adenocarcinoma of the Pancreas (PaC) (data retrieved from [27–29]).

Since emerging evidence is underlining the importance of any kind of substitution as potentially capable to affect protein functionality [19–21], we decided to unbiasedly consider any type of nucleotide substitution in our analysis. So, after retrieving the mutational data, we intentionally generated, for each cancer, a non-stringent list of mutated genes (Supplementary Table S1). Next, to associate those genes with a prediction of clinical-outcome, we took advantage of Precog Meta-Z data [6]. In Precog, Z-scores quantify the correlation or anti-correlation between expression of a specific gene and overall survival of patients. So, we extracted, for each of the tumours analysed, the Z-scores associated with all the genes present in the Precog database (Step 2) and intersected clinical outcome prediction data with our mutational data. By doing so, we divided genes in three classes: *MUT*, genes which were found exclusively mutated, *PRECOG*, genes whose expression only correlated/anti-correlated with a clinical outcome or *MUT + PRECOG*, genes that were mutated and whose expression also correlated/anti-correlated with overall patient survival (Step 3, please refer to the Materials and Methods section for details). Next, for each newly classified gene, we determined the global interactome of its encoded protein (Step 4). To do so we used data derived from the BioGrid [30], a general repository for interaction datasets that combines both physical and genetic interactions. For our analysis, among the full-list of interactors, we considered as cancer-related interactees only those proteins for which the corresponding gene was considered ‘*cancer relevant*’ based on the specific parameters we had implemented (please refer to the Materials and Methods section for threshold parameter setting), and in so doing we generated a virtual interactomic network of cancer-related proteins (Step 5). In these networks we defined as *seeds* those genes with a predicted correlation/anti-correlation with clinical outcome (i.e. proteins of either the *PRECOG* or *MUT + PRECOG* classes) and as *interactors* those genes which were significantly mutated, but not correlated to any clinical outcome (proteins of the *MUT* category). We then determined for each gene, *seed* or *interactor*, a ‘*network* score’ based on the normalized number of mutant interactors its encoded protein had (please refer to the material and methods section for specific score formula and normalization) and we generated, for each cancer, two lists of genes, one containing *seeds* and the other containing *interactors* (Step 6). In both lists, cancer genes were ranked by the capability of their corresponding protein to interact with other mutant proteins (Supplementary Tables S2-S3).

The different steps we have implemented in our approach are detailed in Fig.1 and Supplementary Fig. S1.

### The *network score*

The logic behind the *network score* was to find a way to straightforwardly predict the importance/involvement of a gene in a specific cancer, based primarily on the number of cancer-mutated interactors its corresponding codified protein had.

Before exploring *network score* data and starting to use them to obtain useful information, we assessed the statistical soundness and informativeness of the *score* itself and the validity of our approach.

**Figure 2:**
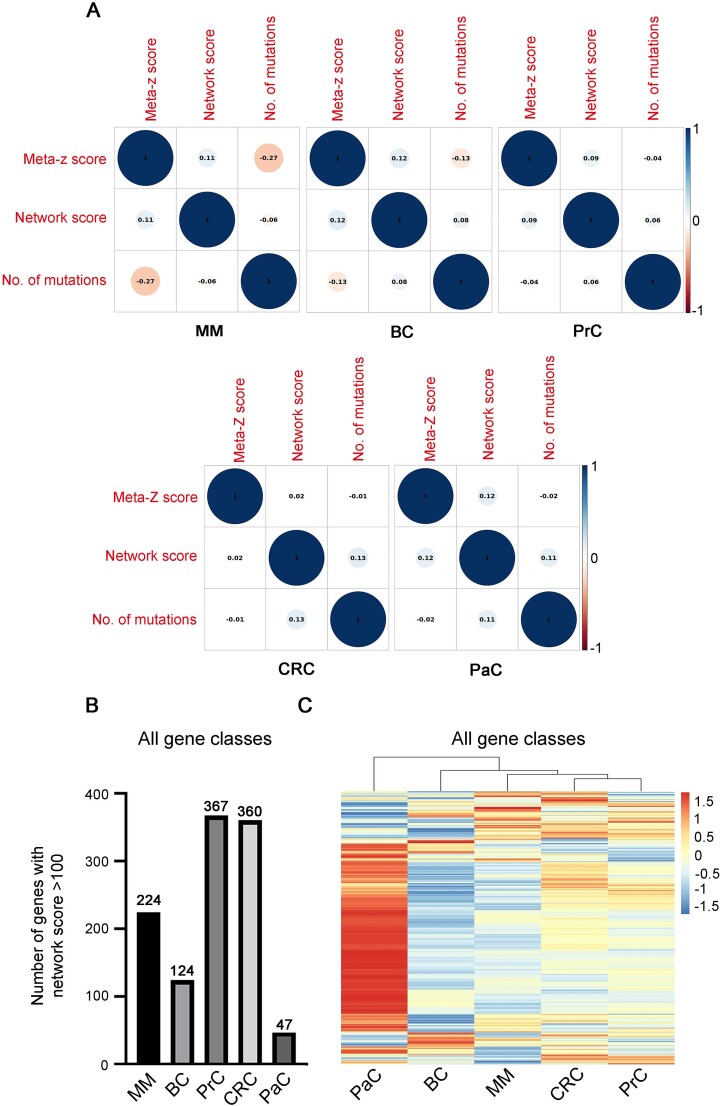
Informativeness of the *network score*. A) Correlation-plot between number of mutations, *Z*- and n*etwork scores* for all gene classes and all the cancers types analysed. Circle dimensions are proportional to absolute correlation coefficient values, which are shown inside each circle. B) Histogram representing for each cancer considered the number of genes with a networks score of over 100. C) Heatmap representing the normalized networks scores for each gene in each cancer considered.MM: Multiple Myeloma; BC: Breast Cancer; PrC: Prostate Cancer; CRC: Colorectal Cancer; PaC: Pancreatic Cancer.

To do so, we analysed correlation between the *network score* attributed to a gene and either the number of total mutations found in that gene or, in the case of *seeds*, its Z-score (Fig. 2A, Supplementary Fig. S2A, top). We found that the *network score* had no correlation with mutation number, suggesting that the information stored in the *network score* is not redundant with that present in the data used to define it. Moreover, *network scores* did not correlate in any way with Z-scores (Fig. 2A and Supplementary Fig. S2A, middle), underlining how the two values actually embed non-redundant information. We also found no linear correlation between Z-scores and number of mutations (Fig. 2A and Supplementary Fig. S2A, bottom), suggesting, as expected, that non-redundant information is also held in each of these variables.

Looking at the overall *network scores* for all gene classes throughout the tumours analysed (Supplementary Tables S2-S3), we found that they were, as expected, higher in those cancers known to have higher mutational rates, i.e. PrC and CRC, and lower in those known to have a lower mutational rate, i.e. BC and PaC (Fig.2B, Supplementary Fig. S2B and S2C) [35]. This is something expected, as the *network score* takes into account the total number of mutated interactors, which is higher when mutational rates increase. For this same reason, the *network score*, as is, is not an absolute value, and quantitative comparisons can be performed only between scores derived from the same cancer.

However, through clustering analysis on *network scores* from the different tumours using Min-Max normalization to ensure comparability, we observed that PrC and CRC clustered closely together. Additionally, MM clustered near BC, while surprisingly, PaC exhibited the highest degree of diversity among the tumour types (Fig. 2C, Supplementary Fig. S2D and S2E). Globally, we found that some genes had generally relatively high scores among most tumours and that some others had high scores only in specific cancer types (Fig.2C, Supplementary Fig. S2D and S2E), suggesting that some genes/pathways might be commonly altered/important in cancer, while some others might be instead cancer-specific.

Overall, these results demonstrate that the *network score* is informative and that it might predict the involvement of specific genes/pathways in cancer.

Next, we performed comparative analyses between our method and similar well-established cancer-gene discovery resources. Being our approach based on a novelly-defined metric, the *network score*, we were unable to perform canonical benchmarking but instead tested if, and to what extent, our pipeline was capable to give results that were comparable to those generated by other cancer-gene discovery methods. Specifically, we used the ‘CancerBrowser’ feature of CancerGeneNet, which derives the most likely paths of causal interactions linking cancer associated genes to cancer phenotypes [36], and the NCG database, a comprehensive catalogue of known and candidate cancer genes derived from cancer sequencing screens [7]. For each tumour considered, we extracted the lists of genes that were either defined or predicted to be cancer-related. Subsequently, we intersected these gene lists with those generated by *CancerHubs*, focusing only on outlier genes, i.e. those genes with *network scores* significantly higher than the mean (refer to the Supplementary Materials and Methods section for details). We found that ~15% of the genes predicted to be cancer-related by either CancerGeneNet or NCG were also outlier genes in *CancerHubs*. This percentage ranged from a peak of 26% for BC to 8% for PaC using CancerGeneNet, and from 23% for PrC to 9% for CRC using the NCG database (Supplementary Table S4). Such percentages underline a level of coherence between the three approaches while also highlighting *CancerHubs*’ potential to enhance and provide novel insights into cancer-gene predictions.

Next, we wanted to determine if genes with higher *network scores* were indeed cancer-related. To do so we performed unbiased pre-ranked Gene Set Enrichment Analysis (GSEA) on the full list of genes ranked by *network score*s for each cancer under consideration. Strikingly, we found that genes with the highest *network scores* defined well-established cancer-related pathways, such as the Myc, mTOR, and WNT/β-Catenin pathways, as well as pathways related to cell cycle and apoptosis (Supplementary Fig. S3, Supplementary Table S5). These findings underscore the reliability of our approach and its ability to define with confidence well-established cancer-related pathways.

Overall, our results show that *CancerHubs* not only accurately predicts the cancer association of genes involved in canonical cancer pathways but also identifies putative novel cancer-related genes.

### *Network scores* define well-known and novel broad cancer protein *hubs*

Having benchmarked our approach, we next wanted to use *network scores* to define cancer related genes.

After evaluating that some high-ranking genes/proteins were shared between cancers (Fig.2C, Supplementary Fig. S2D and S2E), we decided to explore them in more detail. We analysed genes by category (*seed* or *interactor*) and applied a strict threshold by focusing specifically on the top 50 scoring genes of each cancer.

**Figure 3:**
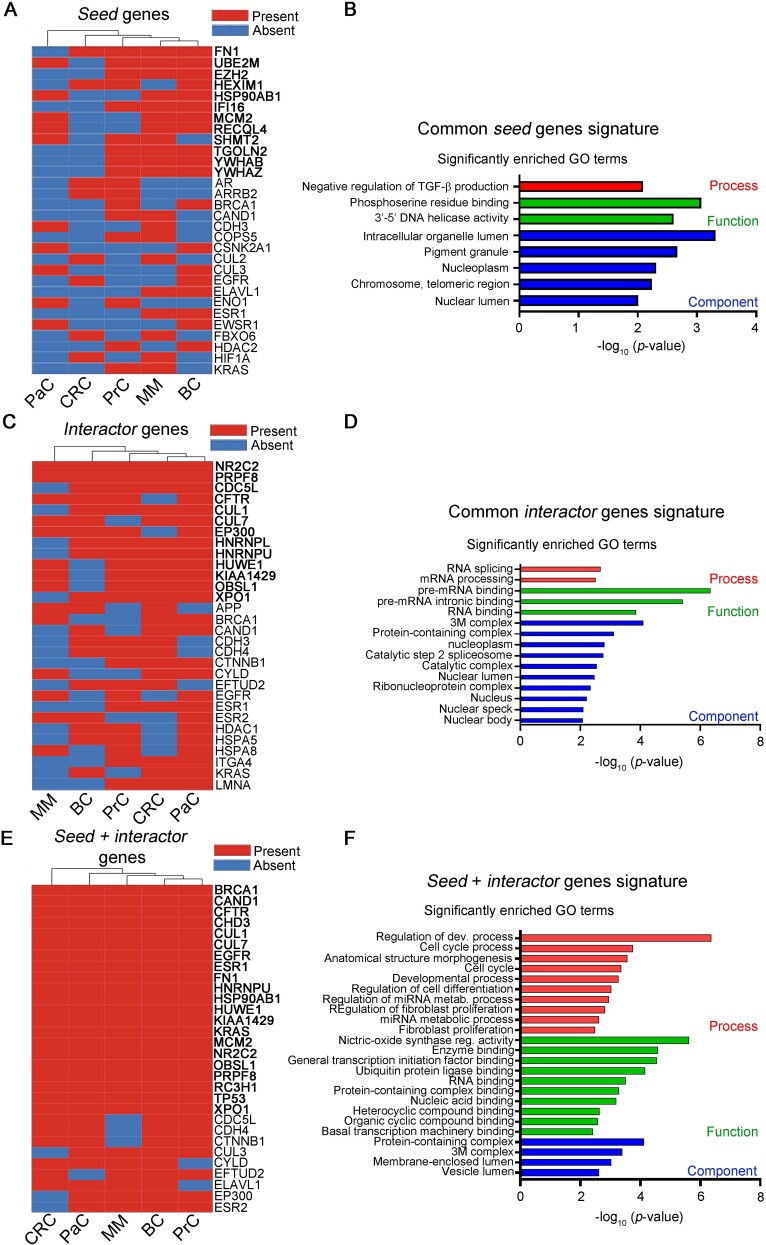
*CancerHubs* predicts novel putative broad cancer protein hubs. A) Heatmap displaying the top 30 genes present in the top 50 network-scoring *seed* genes list of at least 2 out of the 5 cancers analysed. The top 12 genes, identified in at least 3 out of the 5 cancers considered, are highlighted in bold and were utilized for defining GO terms. B) GO terms related to the top 12 common *seed* genes present in at least 3 out of the 5 cancers analysed. C) Heatmap displaying the top 30 genes present in the top 50 network-scoring *interactor* gene lists of at least 3 out of the 5 cancers analysed. The top 13 genes, identified in at least 4 out of the 5 cancers considered, are highlighted in bold and were utilized for defining GO terms. D) GO terms related to the top 13 common *interactor* genes present in at least 4 out of the 5 cancers analysed. E) Heatmap displaying the top 30 genes present in the top 50 network-scoring *seed* or *interactor* gene lists of at least 4 out of the 5 cancers analysed. The top 21 genes, identified in all the 5 cancers considered, are highlighted in bold and were utilized for defining GO terms. F) GO terms related to the top 21 common *seed* + *interactor* genes present in all the 5 cancers analysed.MM: Multiple Myeloma; BC: Breast Cancer; PrC: Prostate Cancer; CRC: Colorectal Cancer; PaC: Pancreatic Cancer.

We started from our *seed* lists (Supplementary Table S2), since we considered *seeds* as the most potentially impacting genes for cancer. Intriguingly, among the top 50 scoring *seed* genes of each cancer, the vast majority were also mutated (45/50 in MM, 41/50 in BC, 50/50 in PrC, 50/50 in CRC and 41/50 in PaC) underlining a clear connection between cancer association and genes that are concomitantly mutated and predicted to influence clinical outcomes. We intersected the *seed* gene list of each cancer with one another, in order to find common-cancer hubs. No gene was shared by all cancers (Supplementary Table S6), something not suprising given their heterogeneity. However, 12 genes were actually common between at least 3 of the 5 cancers analysed (Fig. 3A and Supplementary Fig. S4A, left) suggesting they had a broad involvement in cancer disease.

Among these 12 genes, we found: (i) 10 previously-established oncogenes: namely *FN1*, which is involved in oral squamous cell carcinoma [37], gastric cancer [38, 39] and *BC* [40], *UBE2M*, which is involved in several types of tumours including lung cancer and esophageal squamous cell carcinoma [41], *RECQL4*, whose overexpression is associated with poor prognosis in gastric cancer patients [42], *MCM2*, which is involved in BC, non-small cell lung cancer and hepatocellular carcinoma (HCC) [43], *EZH2*, whose expression is associated with poor prognosis in several malignancies including breast, bladder, endometrial cancer, and melanoma [44], *YWHAB* and *YWHAZ*, encoding components of the 14-3-3 complex, which has long been known to be involved in cancer onset [45], *HSP90AB1*, which is involved in BC [46] and ovarian cancer [47], *IFI16*, whose expression and overexpression correlate with proliferation of CRC cells [48] and with progression of human pancreatic adenocarcinoma, respectively, and *SHMT2*, whose expression promotes tumour growth in BC cells [49] and which is found overexpressed in multiple cancer types [50], (ii) one tumour suppressor: *HEXIM1*, which encodes for a protein involved in the TP53 pathways [51] and which was shown to be involved in BC, PrC, melanoma and acute myeloid leukaemia [52], and (iii) *TGOLN2*, a gene that was previously never reported to be involved in cancer, which encodes for a trans-Golgi network protein.

When we performed this same analysis on the *interactor* protein list (Supplementary Table S3), we found that 13 genes were present in at least 4 of the 5 tumours analysed (Fig. 3C, Supplementary Fig. S4A, right and Supplementary Table S7). Among these, *NR2C2* and *PRPF8* were present in all five cancers. *NR2C2* encodes for a nuclear receptor that was recently shown to function either as an oncosuppressor [53] or as an oncogene [54] based on the specific tumour type analysed, while *PRPF8* encodes for a protein involved in mRNA splicing and its expression was recently demonstrated to increase the aggressiveness of HCC [55].

The remainder genes were: *KIAA1429*, a well-established oncogene [56]; *CFTR*, a tumour suppressor in intestinal cancer [57]; *HUWE1*, *CUL1*, *CUL7*, and *OBSL1*, encoding proteins involved in ubiquitin-ligase binding, a pathway often deregulated in cancers; *EP300*, which encodes for a histone acetyl-transferase and is a well-established tumour suppressor [58]; *XPO1*, which has a broad cancer involvement [59]; *CDC5L*, which is a well-established oncogene [60, 61]; and *HNRNPL* and *HNRNPU*, which encode for proteins involved in RNA metabolism, but whose involvement in cancer is just starting to be elucidated.

Overall these results indicate not only that our approach efficiently defines already established cancer-related genes, but, more importantly, that it can predict novel cancer hubs, e.g. *TGOLN2*, *HNRNPL*, and *NHRNPU*.

Intriguingly, canonical cancer-related genes such as *KRAS*, *TP53*, *PTEN*, *MYC*, *EGFR*, *MDM2* and *BRCA1* did not fall among our top-scoring s*eed* or *interactor* genes. However, when we further explored our data by merging the top-50 scoring *seed* and *interactor* genes of each cancer, we found that such genes were present in at least three out of the five tumours analysed (Fig. 3E and Supplementary Table S8). This suggests that our method primarily identifies components of pathways that are utilized by canonical cancer-related genes rather than the canonical cancer-related genes themselves, again underlining the novelty of our approach.

Next, in order to define broad-cancer-related pathways, we searched for shared features between the lists of common *seeds* and *interactors* by performing Gene Ontology (GO) analysis either separately on the two lists of genes, or by merging the lists together (Fig. 3E and Supplementary Fig. S4A). On the seed list we found a significant correlation with negative regulation of TGF-β signalling, DNA replication and intracellular organelle localization/components (Fig.3B), on the interactor list we found significant connection with RNA splicing (Fig. 3D), while on the merged list we found correlation with cell cycle control, RNA metabolism and transcription (Fig.3F and Supplementary Fig. S4B).

These results suggest that regulation of cell growth and of gene expression are among the pathways most globally altered in cancer, as previously suggested [62], and confirm the soundness of our approach also in predicting broad-cancer-related pathways.

### *Network scores* define well-known and novel cancer-specific protein *hubs*

Next, we aimed to determine cancer-specific protein hubs.

**Figure 4:**
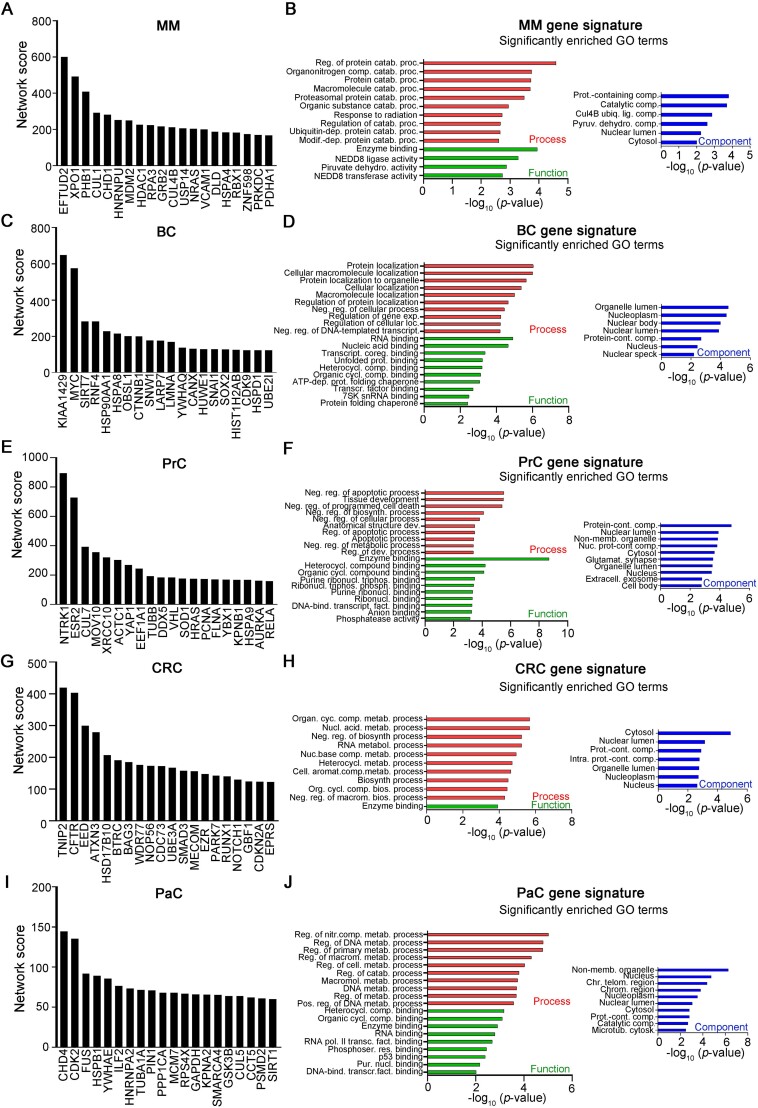
*CancerHubs* predicts novel putative cancer-specific *seed* protein hubs. Histograms representing the network scores of the top-20 (A) MM-specific, (C) BC-specific, (E) PrC-specific, (G) CRC-specific and (I) PaC-specific seed genes. GO terms related to the top-20 (B) MM-specific, (D) BC-specific, (F) PrC-specific, (H) CRC-specific and (J) PaC-specific seed genes. If more than 10, only top-10 GO terms per each ontology of each cancer are shown. MM: Multiple Myeloma; BC: Breast Cancer; PrC: Prostate Cancer; CRC: Colorectal Cancer; PaC: Pancreatic Cancer.

Again, we focused only on the top 50 ranked-genes of each cancer and, starting from the *seed* gene list, we determined those genes which were present only in one out of the five cancers considered. The top 20 cancer-specific *seed* hubs per each cancer are shown in Fig. 4 and Supplementary Table S9.

Such genes included well-established cancer-related genes, like *XPO1*, *MDM2* and *NRAS* for MM (Fig. 4A), *MYC*, *SIRT7*, and *CDK9* for BC (Fig. 4C), *ESR2*, *YAP1*, and *HRAS* for PrC (Fig. 4E), *SMAD3*, *RUNX1*, and *NOTCH1* for CRC (Fig. 4G) and *CHD4*, *CDK2*, and *MCM7* for PaC (Fig. 4I), but also genes whose involvement in the specific cancer in which we defined them as hubs was never assessed before. Examples include *EFTUD2* and *PHB* for MM (Fig. 4A), *OBSL1* and *HIST1H2AB* for BC (Fig. 4B), *CUL7* and *MOV10* for PrC (Fig. 4C), *GBF1* and *EPRS* for CRC (Fig. 4D), and *TUBA1A* and *PSMD2* for PaC (Fig. 4E).

Next, we focused on the *interactor* protein lists. Cancer-specific *interactor* hubs are listed in Supplementary Fig. S5 and Supplementary Table S10. Again, for each cancer we found a list of well-established cancer-related genes, e.g. *RELA* and *HDAC4* in MM (Supplementary Fig. S5A), *MYH9* and *AKT1* in BC (Supplementary Fig. S5C), *ATXN3* in PrC (Supplementary Fig. S5E), *RECQL4* and *EZH2* in CRC (Fig. S5G), and *FN1* and *HIF1A* in PaC (Supplementary Fig. S5I), but also a set of novelly defined cancer-related genes, including *CSNK2A1* and *FBXW11* in MM (Supplementary Fig. S5A), *HIST1H3A*, *HIST1H2BB* and *GOLGA2* in BC (Supplementary Fig. S5C), *PIH1D1* in CRC (Supplementary Fig. S5G), and *TMPO* in PaC (Supplementary Fig. S5I). These results, once again, suggest how *CancerHubs* can be a valid approach to predict novel cancer-related genes.

In order to determine cancer-specific hub-associated networks, we performed GO analysis on the *seed*-hub lists. We found that (i) MM-specific *seeds* correlated with protein homeostatis and degradation and with protein complex enzymatic activity (Fig. 4B), a result which is in line with the patophysiology of MM, a malignancy involving highly-secreting plasmacells, which deeply rely in a tight regulation of protein homeostasis, (ii) BC *seeds* mostly correlated with transcription regulation (Fig. 4D), a results which is in agreement with recently published results [63], (iii) PrC *seeds* specifically correlated with regulation of apoptotic responses and DNA metabolism (Fig. 4F), two pathways often altered in cancer, (iv) CRC genes correlated with RNA metabolism [64] and biosynthetic enzymatic processes [65] (Fig. 4H), and (v) PaC genes mainly correlated with DNA/RNA metabolism and cytoskeleton organization (Fig. 4J), in agreement with recent published data [66–68]. These results suggest that specific pathways are actually differentially modulated/altered in different cancer, possibly due to the different nature of the cancers themselves.

Intriguingly, despite involving different genes, all cancer-specific *seed* hubs had GO signatures correlated to organelle components, indicating the importance of correct organelle functionality/regulation for maintaining cellular homeostasis and thus preventing cancer.

Next, in order to define cancer-specific *interactor* related pathways, we performed GO analysis on cancer-specific *interactor* hubs (Supplementary Fig. S5 and Supplementary Table S10) and found that (i) MM genes correlated with transcription regulation, and, once again, with protein homeostasis (Supplementary Fig. S5B), confirming the results obtained analysing *seed*-hub networks, (ii) BC hubs correlated with cell cycle regulation, potassium channel activity and cell junction components (Supplementary Fig. S5D), in accordance with studies correlating functionality of these pathways specifically with BC severity [69], (iii) PrC genes correlated with ER stress responses and ubiquitin activity (Supplementary Fig. S5F), in agreement with previously published work [70], (iv) CRC hubs significantly correlated with methyltransferase activity (Supplementary Fig. S5H), which is well-known to be associated with cancer onset, and (v) PaC significantly correlated with transcriptional regulation and, intriguingly, with pathways involved with symbiont interaction (Supplementary Fig. S5J), a result that is however in agreement with recent data depicting the pancreas as a homoestatic regulator of the gut microbiota [71]. Again, these data suggest how different cancers rely on preferential modulation/alteration of different pathways. Altogether our results indicate that *CancerHubs* is functional in defining both cancer-specific hubs and cancer-specific hub-related networks and hence could be a useful tool for discovering novel cancer associated genes/pathways.

### Validation of *CancerHubs* predictions: TGOLN2 has tumour suppressor-like properties is BC, MM, and PrC and EFTUD2 behaves as an oncogene in MM

Next, we wanted to actually validate the predictions provided by *CancerHubs* regarding novel cancer genes.

Starting from the *seed* lists, we selected one broad cancer tumour suppressor-like gene and one cancer-specific gene with oncogenic potential to validate, namely *TGOLN2* and *EFTUD2*. The first encodes for a trans-Golgi protein involved in exocytic vesicle formation, which is predicted by Precog to be a tumour suppressor in BC, MM and PrC; the second encodes for a component of the spliceosome, and is predicted to be a novel oncogene hub in MM.

**Figure 5:**
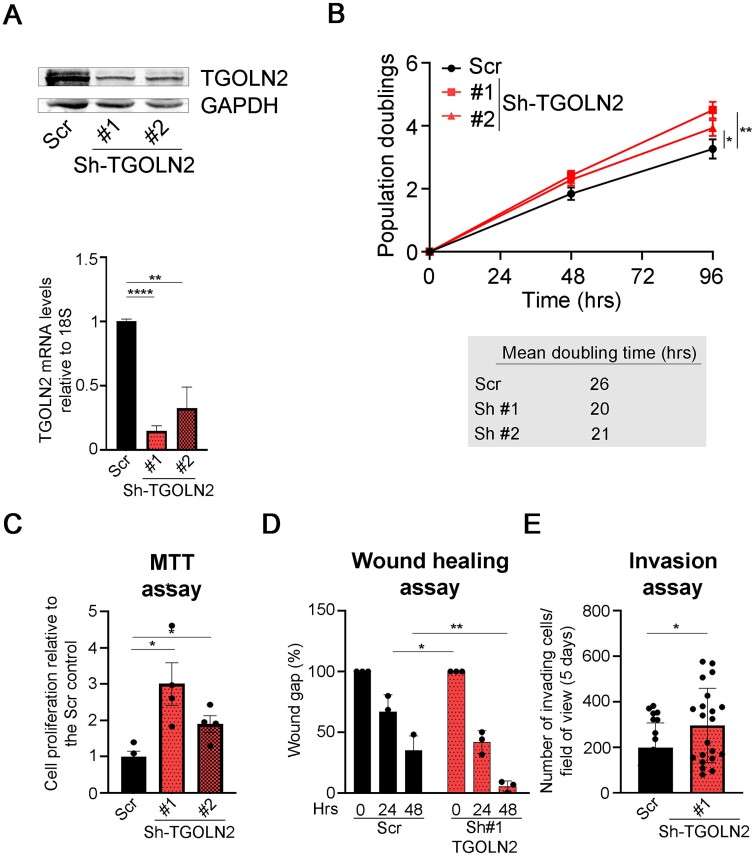
TGOLN2 has tumour suppressor-like properties in in vitro models of BC. A) TGOLN2 levels in the MCF-7 BC cell line with *TGOLN2* downmodulating constructs. Top) Western blot showing the levels of the TGOLN2 protein in the MCF-7 BC cell line expressing either a control plasmid (Scr) or Sh-RNA constructs targeting *TGOLN2* (sh-TGOLN2 #1 and #2). GAPDH was used as loading control. Bottom) RT-qPCR showing mRNA levels of *TGOLN2* in the same cell lines. B) Proliferation assay in MCF-7 BC cells with *TGOLN2* downmodulation. Same cell lines as in A) were used to perform a proliferation assays (top) and assess doubling times (bottom). C) MTT assay of MCF-7 BC cell lines with *TGOLN2* downmodulation. Same cells as in A) were used to assess metabolic activity through MTT assays. Histograms represent the mean +/- SEM of four independent experiments. D) Migration assay of MCF-7 BC cell lines with *TGOLN2* downmodulation. The migration capacity of MCF-7 scr and sh-*TGOLN2* #1 BC cells was assessed through scratch assays. E) Invasion assay of MCF-7 BC cell lines with *TGOLN2* downmodulation. Same cells as in D) were used to assess invasion capacity using matrigel-coated transwell assays. Points represent cell counts/fields of view of 21 fields of view. Except where otherwise stated, all histograms represent the mean +/- SD of a minimum of three independent experiments. Statistical *P*-values were calculated using unpaired *t*-tests. ns: *P* > 0.05; ^*^: *P* < 0.05; ^*^^*^: *P* < 0.01; ^*^^*^^*^: *P* < 0.001; ^*^^*^^*^^*^: *P* < 0.0001. Blots are representative of three independent experiments.

At first, to formally demonstrate that TGOLN2 had tumour suppressor properties, we selected cell lines with high TGOLN2 levels deriving from tumours for which *TGOLN2* was predicted to be a cancer hub. Taking advantage of the Human Protein Atlas (https://www.proteinatlas.org/), we selected the MCF-7 cell line, derived from BC, the LP-1 cell line, derived from MM and the DU-145 cell line, derived from PrC and downmodulated the gene using two different shRNAs (Fig. 5A, Supplementary Fig. S6A and S6D). We then monitored cell proliferation migration and invasion in control cells and in cells with reduced levels of TGOLN2. The two shRNAs both downmodulated *TGOLN2* mRNA levels but to different extents (Fig. 5A and Supplementary Fig. S6A and S6D). When we monitored cell proliferation, we found that *TGOLN2* downmodulation actually increased proliferation in all the cell lines analysed (Fig. 5B and Supplementary Fig. S6B and S6E), something expected when downmodulating a tumour suppressor-like gene. Intriguingly the effect was proportional to *TGOLN2* downmodulation (Fig. 5A and Supplementary Fig. S6A and S6D), confirming that cell proliferation capability is actually inversely correlated with TGOLN2 levels. To confirm this result we performed MTT assays, which monitor the metabolic activity of cells. We found not only that *TGOLN2* donwmodulation favoured an increase in cell metabolism (Fig. 5C and Supplementary Fig. S6C), but also that such increase was again proportional with *TGOLN2* downmodulation, strenghtening the idea that TGOLN2 could actually function as a tumour suppressor. When we assessed cell migration and invasion capabilities using a transwell approach (Fig. 5D-E and Supplementary Fig. S7A-B) we confirmed our results: *TGOLN2* downmodulation correlated with cells acquiring the ability to migrate (Fig. 5D, Supplementary Fig. S7A) and invade (Fig. 5E and Supplementary Fig. S7B). Overall, we showed that TGOLN2 has tumour suppressor-like properties in BC, MM and PrC.

**Figure 6:**
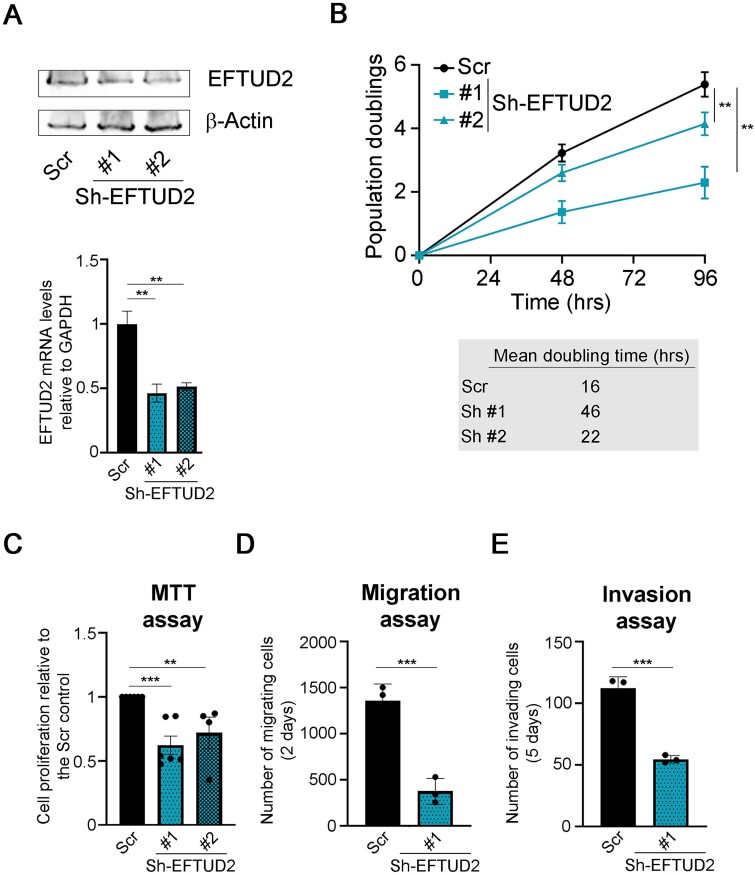
EFTUD2 has oncogenic properties in in vitro models of MM. A) EFTUD2 levels in the RPMI-8226 MM cell line with *EFTUD2* downmodulating constructs. Top) Western blot showing the levels of the EFTUD2 protein in the RPMI-8226 MM cell line expressing either a control plasmid (Scr) or Sh-RNA constructs targeting *EFTUD2* (sh-EFTUD2 #1 and #2). β-actin was used as loading control. Bottom) RT-qPCR showing mRNA levels of *EFTUD2* in the same cell lines. B) Proliferation assay in MM RPMI-8226 cells with *EFTUD2* downmodulation. Same cell lines as in A) were used to perform a proliferation assays (top) and assess doubling times (bottom). C) MTT assay of RPMI-8226 MM cell lines with *EFTUD2* downmodulation. Same cells as in A) were used to assess metabolic activity through MTT assays. Histograms represent the mean +/- SEM of a minimum of four independent experiments. D) Migration assay of RPMI-8226 MM cell lines with *EFTUD2* downmodulation. The migration capacity of RPMI-8226 MM control and sh-*EFTUD2* #1 cells was assessed through transwell assays. E) Invasion assay of RPMI-8226 MM cell lines with *EFTUD2* downmodulation. Same cells as in D) were used to assess invasion capacity using matrigel-coated transwell assays. Except where otherwise stated, all histograms represent the mean +/- SD of a minimum of three independent experiments. Statistical *P*-values were calculated using unpaired *t*-tests. ns: *P* > 0.05; ^*^^*^: *P* < 0.01; ^*^^*^^*^: *P* < 0.001; ^*^^*^^*^^*^: *P* < 0.0001. Blots are representative of three independent experiments.

Next, we focused on *EFTUD2*. We selected a MM cell line with high EFTUD2 levels, namely the RPMI-8226 cell line, and downmodulated the gene using two different shRNAs (Fig. 6A). We found that downmodulation of *EFTUD2* levels actually correlated with reduced proliferation (Fig. 6B), and that the extent of such reductions correlated with the reduction of EFTUD2 levels (Fig. 6A), confirming the putative oncogenic role of EFTUD2 in MM. Next we performed MTT assays, as described above, and found that *EFTUD2* downmodulation correlated with decreased metabolic activity (Fig. 6C). When we performed migration and invasion assays we found that reduced EFTUD2 levels actually correlated with decreased migration (Fig. 6D and Supplementary Fig. S7C) and invasion (Fig. 6E and Supplementary Fig. S7D), fully confirming that *EFTUD2* is indeed a *bona fide* gene with oncogenic functions in MM.

Overall, these results validate the efficacy of *CancerHubs* predictions and establish *TGOLN2* as a novel broad cancer *hub* with tumour suppressor-like properties and *EFTUD2* as a novel MM *hub* with oncogenic properties.

## Conclusion

Here, we presented a novel method to define cancer-related genes: *CancerHubs*.

Using this approach, genes related to cancer are given a score and are ranked not on the frequency of their mutations nor on the correlation between their expression and clinical outcome, but on the number of mutated interactors their corresponding encoded protein has. This approach is based on two assumptions: (i) in cancer, proteins involved in pathways that are critical for maintaining cell homeostasis are frequently mutated in modules and (ii) proteins involved in the same pathway often interact with one another. Moreover, it relies on a straight-forward rationale: cancer-related proteins with a high impact in cancer should preferentially fall in one (or more) interaction network(s) composed by several other mutated proteins. Specifically, the more a protein interacts with mutated proteins in a specific cancer type, the more that protein should be involved in that cancer.

Our approach is a clear advance over existing state-of-the-art methods. First of all, because it takes into account and merges with interactomics two of the most common and widely-used approaches to predict cancer-related genes, namely mutation analysis and clinical outcome predictions. Secondly, because by considering any type of mutation as potentially deleterious for downstream protein functionality, it broadens the spectra of genes to be analysed.

The analyses we presented in this work focused only on the top-50 scoring genes per each cancer. This was a conscious thought as our idea was to deal with a short and very strictly-filtered gene list. In so doing, we showed that *CancerHubs* has the power not only to define well-established oncogenes/tumour suppressors as cancer hubs, but also to predict novel, previously unexplored, cancer players.

Here, we validated two selected cancer-related genes from the *seed* lists: *TGOLN2*, a new broad cancer *hub* with tumour suppressor-like properties and *EFTUD2*, a novel MM-specific hub with oncogenic potential.

We demonstrated TGOLN2 involvement in BC, MM and PrC, but can actually speculate on a much broader involvement. The fact that *TGOLN2* expression was recently shown to correlate with irinotecan sensitivity in CRC [72] and cisplatin resistance in lung adenocarcinioma [73] supports this idea and confirms the reliability of *CancerHubs* predictions.

Among the list of cancer-specific *seeds* we validated *EFTUD2* in MM. Intriguingly, despite *EFTUD2* was found as a *seed* only in MM, the protein was present as a top scorer *interactor* hub also in BC, PrC, and CRC, suggesting that it might actually possess a broader cancer-related role. Indeed, EFTUD2 is also identified as a candidate cancer driver by the NCG database and recent evidence pointed at an oncogenic role for EFTUD2 in both BC [74] and CRC [75] but also in HCC [76], again strengthening the idea that our approach is a valid method to predict cancer-related genes, and underlining the importance also of *interactor* hubs.

Besides *TGOLN2* and *EFTUD2*, our method predicted several other broad cancer or cancer-specific hubs.

*UBE2M*, *FN1*, and *RECQL4*, for example, fell among our top 12 common-cancer *seed* hubs.

While UBE2M, is only recently starting to be envisaged as a cancer player [77], *FN1* and *RECQL4* are two well-known oncogenes. Intriguingly, however, despite C*ancerHubs* predicted them to be involved in MM (they both fell in the top-15 MM hub genes), this was never experimentally confirmed neither by us nor by others, underlining once again the predictive utility of our approach.

Another exemplificative case is that of *KIAA1429*. Just like *EFTUD2* for MM, *KIAA1429* was found to be a cancer-specific *seed* hub for BC, but also a broad cancer *interactor* hub, pointing to a broader cancer role for KIAA1429. Recent work actually confirms this, as KIAA1429 involvement in BC was thoroughly demonstrated [78–81], as well as its involvement in other malignancies, including B cell lymphoma [82], HCC [83] and liver cancer [84]. Again, these considerations confirm the reliability of the predictions of *CancerHubs* and suggest that *seed* and *interactor* lists are actually equally usefull and predictive for defining cancer-related genes. Intriguingly, despite having significantly high network scores, some well-established oncogenes and tumour suppressors, like *KRAS*, *TP53*, *PTEN*, *MYC*, *EGFR*, *MDM2* and *BRCA1*, did not fall among the top-50 scoring genes shared by the cancers analysed. This suggests that (i) *CancerHubs* primarily defines genes that work in pathways employed by canonical cancer genes rather than the canonical cancer genes themselves and (ii) focusing only on the top-50 scoring genes might be too restrictive, potentially causing some putative novel cancer-related genes to be overlooked. Therefore, future analyses should use wider thresholds, which could help identify additional and entirely new cancer hubs.

We also predict future implementations for our method and these will include: (i) addition of new representative datasets for the cancers we have already included in our analysis, in order to strengthen the significance of the gene lists we produce, (ii) expansion of our protein–protein interaction data using also other repositories, in order to strengthen the reliability of the network scores generated, (iii) addition of clinical data derived from other cancers, in order to define more comprehensive and precise gene lists, specifically, we plan to add other liquid tumours, and (iv) merging of other clinical outcome data in order to strengthen our predictions regarding oncogene or tumour suppressor functions. In conclusion, *CancerHubs* is a novel approach to predict gene involvement in cancer. By ranking cancer-related genes based on the number of mutant interactors their encoded proteins have, this approach establishes hubs of mutated proteins with a putative relevance for cancer. This method globally improves the detection of cancer genes, as we show that it is capable to effectively predict protein hubs that were previously never found related to cancer.

**Key Points**

- *CancerHubs* combines, for the first time, unbiased mutational data, clinical outcome predictions and interactomic data in order to define if, and to what extent, cancer-related proteins are part of broader cancer-mutated networks.
- The *CancerHubs* method relies on the newly defined *network score*, a metric which reflects gene involvement/impact in a specific cancer based on the number of mutated interactors its encoded protein has.
- Ranking of genes/proteins based on *network scores* reveals novel broad cancer and cancer-specific protein hubs.
- By validating *CancerHub*s predictions we demonstrated that TGOLN2 is a protein with tumour suppressor features in Multiple Myeloma, Breast and Prostate Cancer and that EFTUD2 has an oncogene-like behaviour in Multiple Myeloma.

## Supplementary Material

Supplementary_figures_Briefings_in_bioinfo_30_10_24_bribio_bbae635

Table_S1_bribio_bbae635

Table_S2_bribio_bbae635

Table_S3_bribio_bbae635

Table_S4_bribio_bbae635

Table_S5_bribio_bbae635

Table_S6_bribio_bbae635

Table_S7_bribio_bbae635

Table_S8_bribio_bbae635

Table_S9_bribio_bbae635

Table_S10_bribio_bbae635

Supplementary_Materials_and_methods_ok_bribio_bbae635

## Data Availability

All data generated or analysed during this study are available on GitHub at https://github.com/ingmbioinfo/cancerhubs.
