# Supplementary material for: CancerHubs: a systematic data mining and elaboration approach for identifying novel cancer-related protein interaction hubs
Source: Brief Bioinform. 2024 Dec 7;26(1):bbae635. doi: 10.1093/bib/bbae635 (PMC11631132; doi:10.1093/bib/bbae635)
Supplement: Supplementary_figures_Briefings_in_bioinfo_30_10_24_bribio_bbae635 [file supplementary_figures_briefings_in_bioinfo_30_10_24_bribio_bbae635.docx]

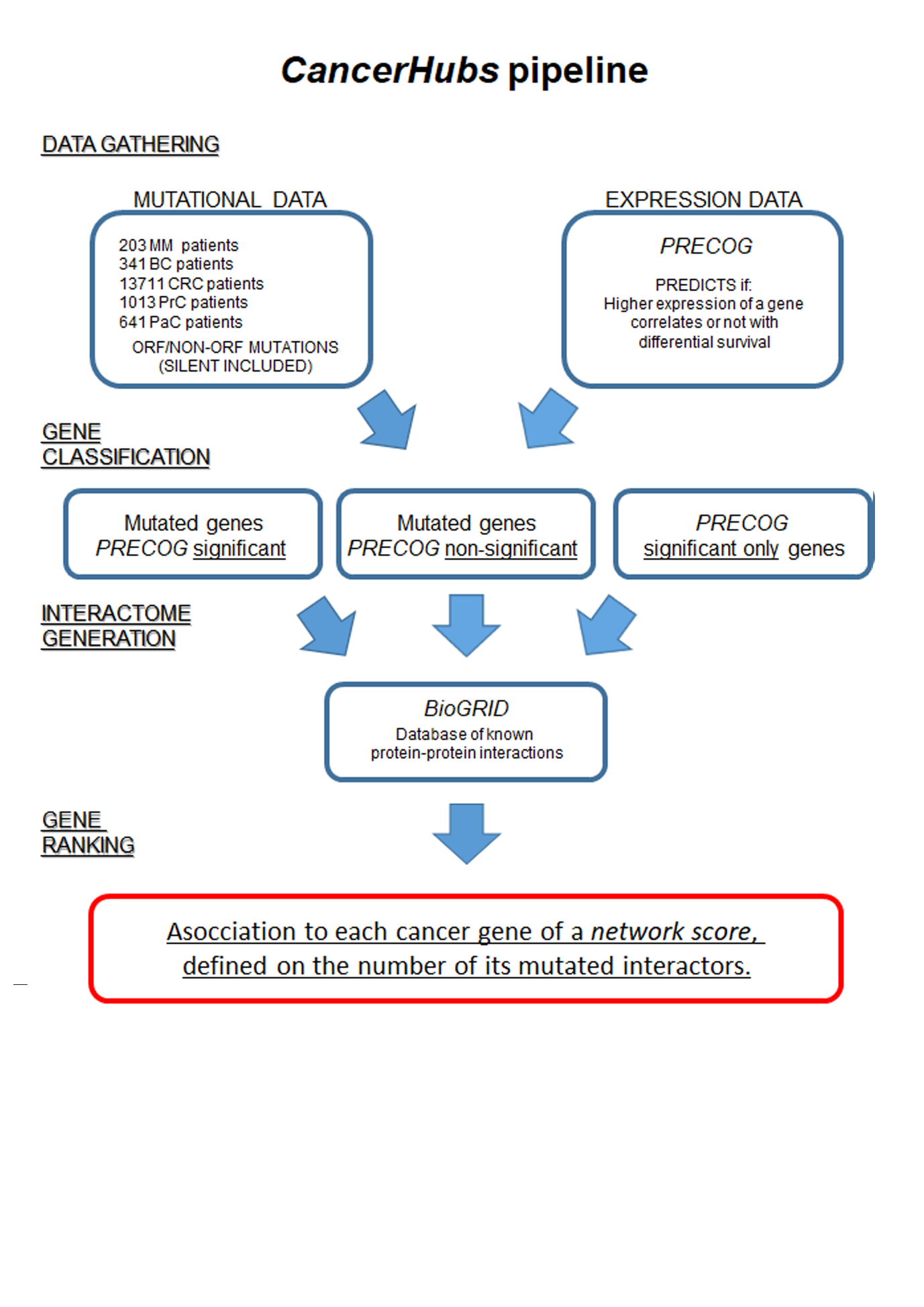


**Supplementary Figure S1. *CancerHubs* pipeline.**

The *CancerHubs* pipeline is composed by a “Data gathering” phase, in which databases are selected and data is retrieved, a “Classification” phase in which genes are classified based on their mutational status and on their correlation/anti-correlation with clinical outcome prediction, an “Interactome generation” phase, in which cancer-related gene interactomes are produced and a “gene ranking” phase in which each gene is given a score based on the number of its “mutated” interactors.

MM: Multiple Myeloma; BC: Breast Cancer; PrC: Prostate Cancer; CRC: Colorectal Cancer; PaC: Pancreatic Cancer.


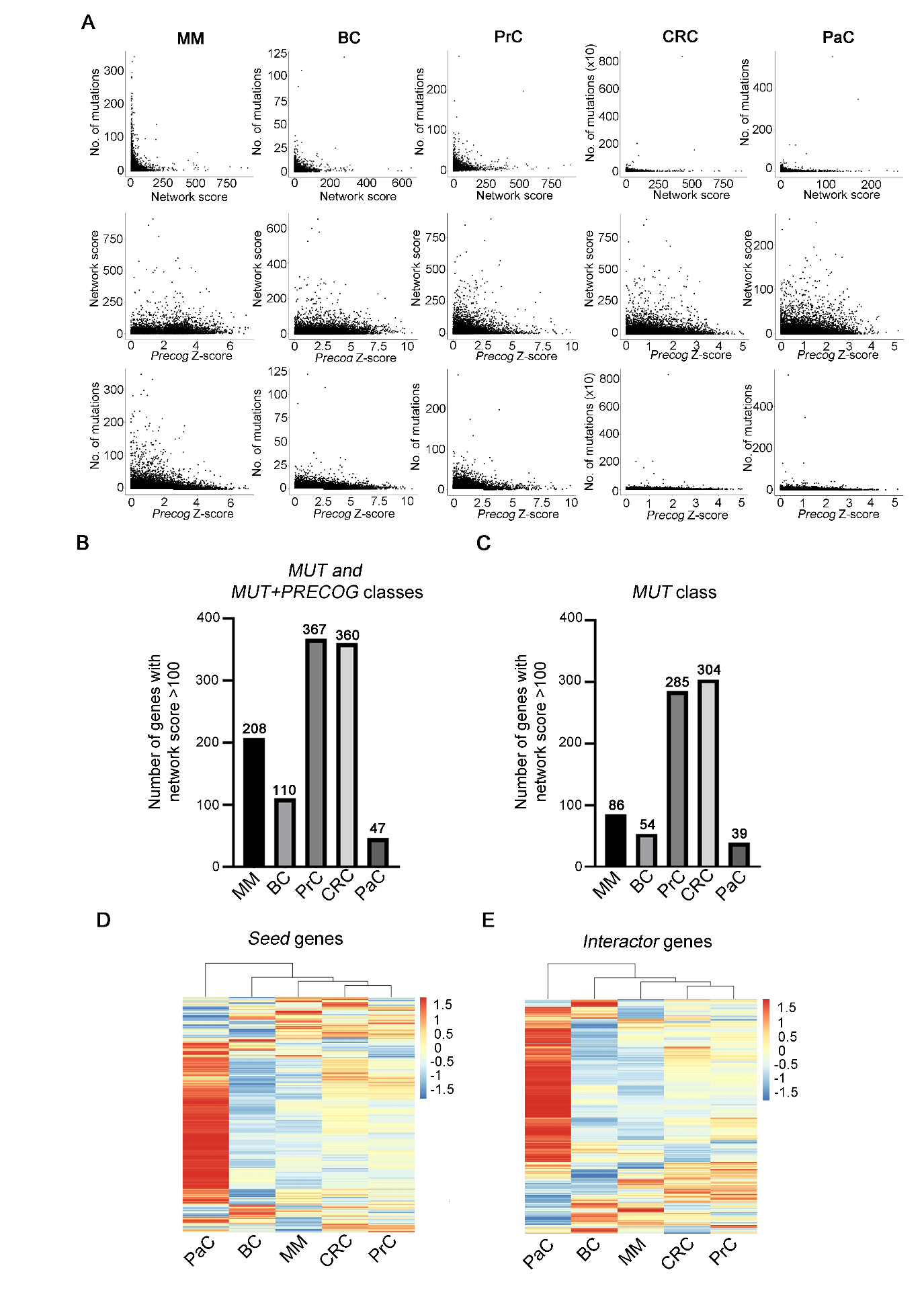


**Supplementary Figure S2. *Network scores* are not redundant with mutation number or Z-scores and are higher in cancers known to have a higher number of gene mutations.**

A) Dot plots representing correlation between number of mutations, Z-scores, and *network scores* for all gene classes and all cancers analysed.

B) Histogram representing for each cancer considered the number of *MUT* or *MUT+PRECOG* class genes with a *network score* of over 100.

C) Same as in C but only *MUT* class genes are shown.

D) Heatmap representing the normalized networks scores for each *seed* gene in each cancer considered.

E) Same as in D but *interactor* genes are shown.

MM: Multiple Myeloma; BC: Breast Cancer; PrC: Prostate Cancer; CRC: Colorectal Cancer; PaC: Pancreatic Cancer.


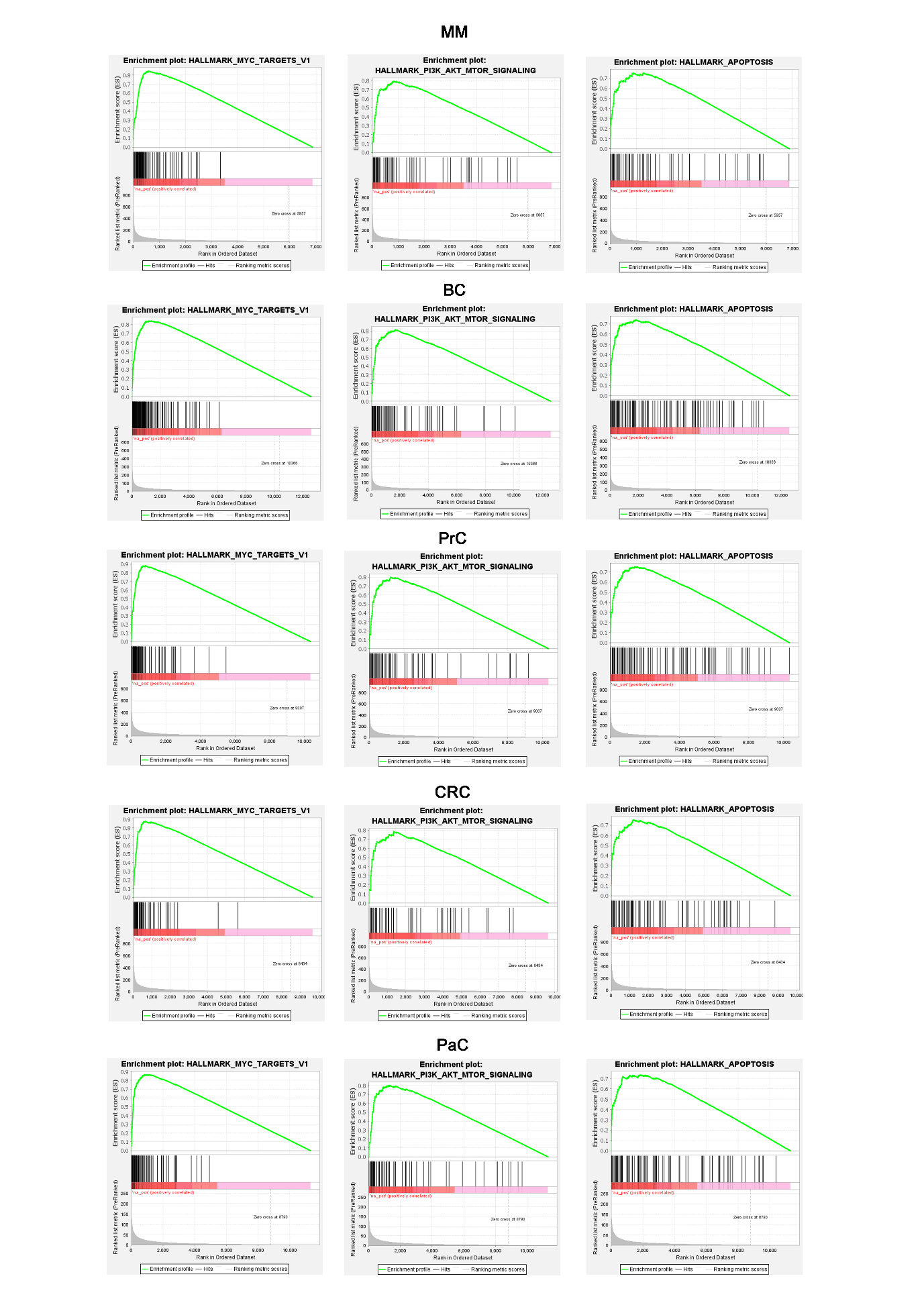


**Supplementary Figure S3. GSEA reveals correlation between high network-scoring genes and well-established cancer related pathways.**

For each cancer considered Enriched Gene Sets associated to cancer-related pathways are shown. GSEA was performed on the full list of genes (*seeds* + *interactors*) pre-ranked by *network score*. Gene sets were considered significantly enriched when both Nominal *p-values* and FDR *q-values* were below 0.05. MM: Multiple Myeloma; BC: Breast Cancer; PrC: Prostate Cancer; CRC: Colorectal Cancer; PaC: Pancreatic Cancer.

**
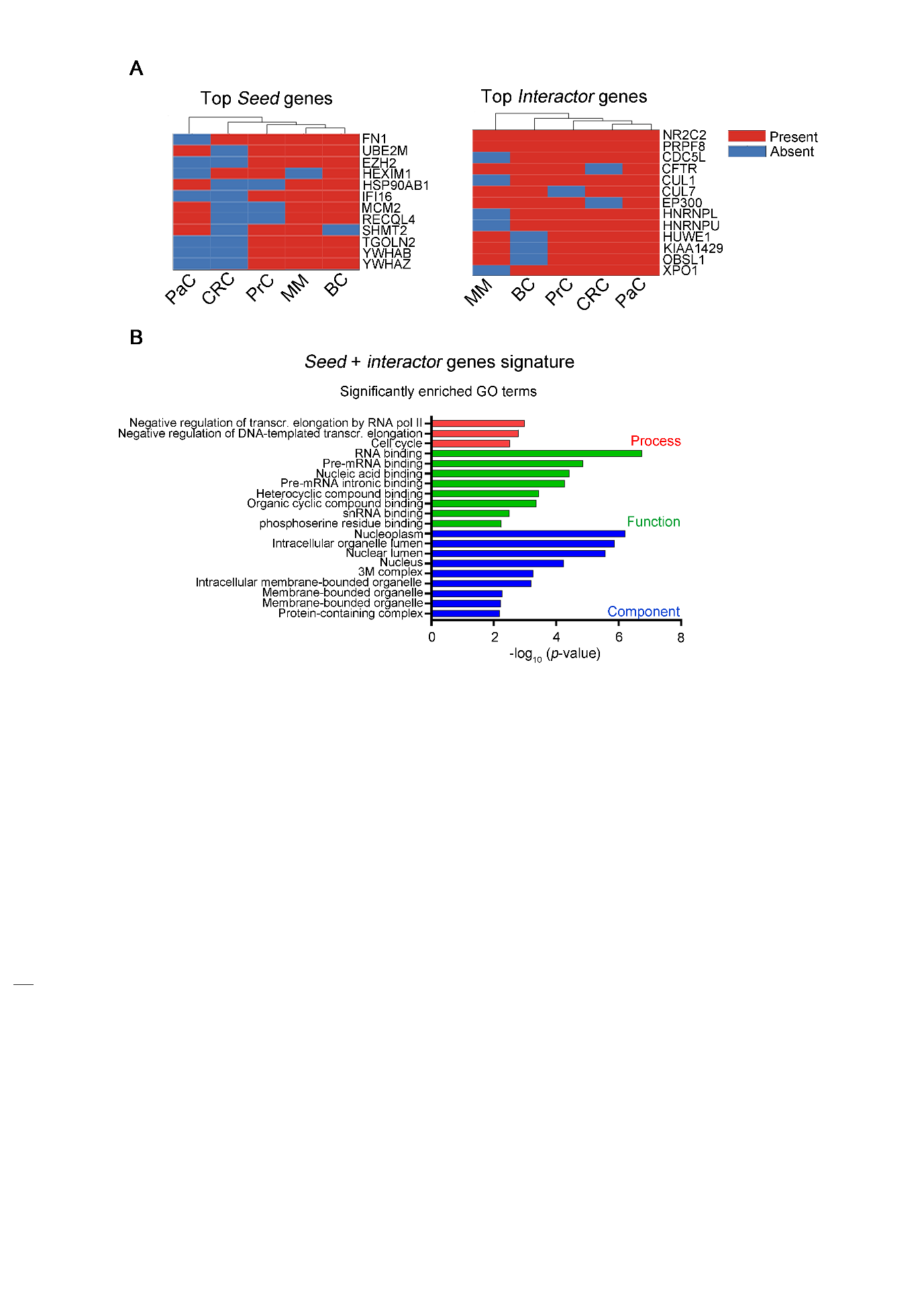
**

**Supplementary Figure S4. Broad-cancer *seed* and *interactor* genes define specific GO terms.**

A) Heatmaps displaying the top broad-cancer *seed* or *interactor* genes.

Left, top 12 *seed* genes identified in at least 3 out of the 5 cancers considered. Right, top 13 *interactor* genes, identified in at least 3 out of the 5 cancers considered.

B) GO terms related to the top 12 common *seed* and top 13 common *interactor* genes shown in A).

MM: Multiple Myeloma; BC: Breast Cancer; PrC: Prostate Cancer; CRC: Colorectal Cancer; PaC: Pancreatic Cancer.


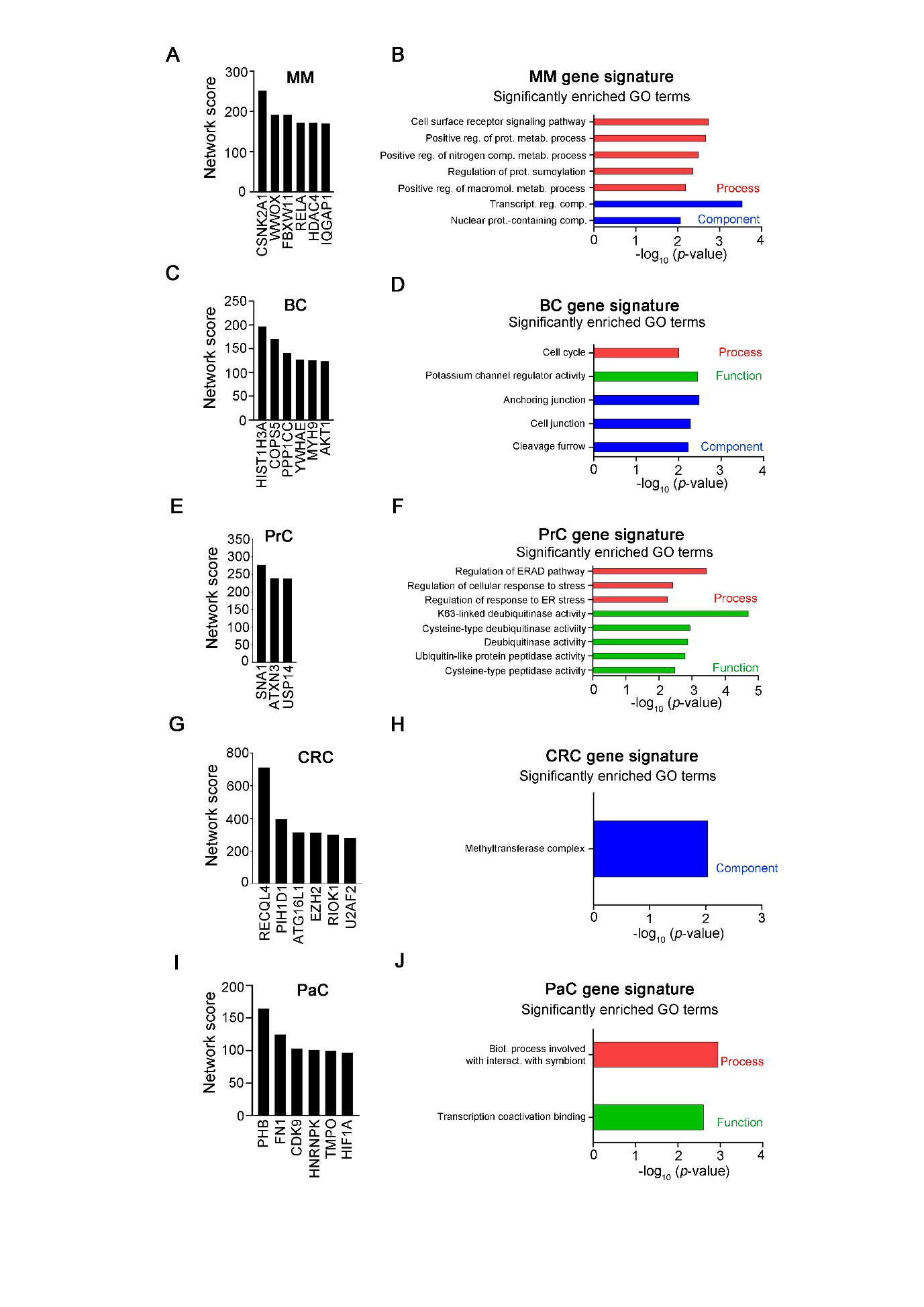


**Supplementary Figure S5. *CancerHubs* can predict novel and well-established cancer-specific *interactor* protein hubs.**

Histograms representing the *network scores* of the top-6 (A) MM-specific, (C) BC-specific, (E) PrC-specific, (G) CRC-specific and (I) PaC-specific *interactor* genes. Where less than 6, the maximum number of cancer-specific genes is shown.

GO terms related to the top-6 (B) MM-specific, (D) BC-specific, (F) PrC-specific, (H) CRC-specific and (J) PaC-specific *interactor* genes. Where less than 6, the maximum number of cancer-specific genes were considered.

MM: Multiple Myeloma; BC: Breast Cancer; PrC: Prostate Cancer; CRC: Colorectal Cancer; PaC: Pancreatic Cancer.

**
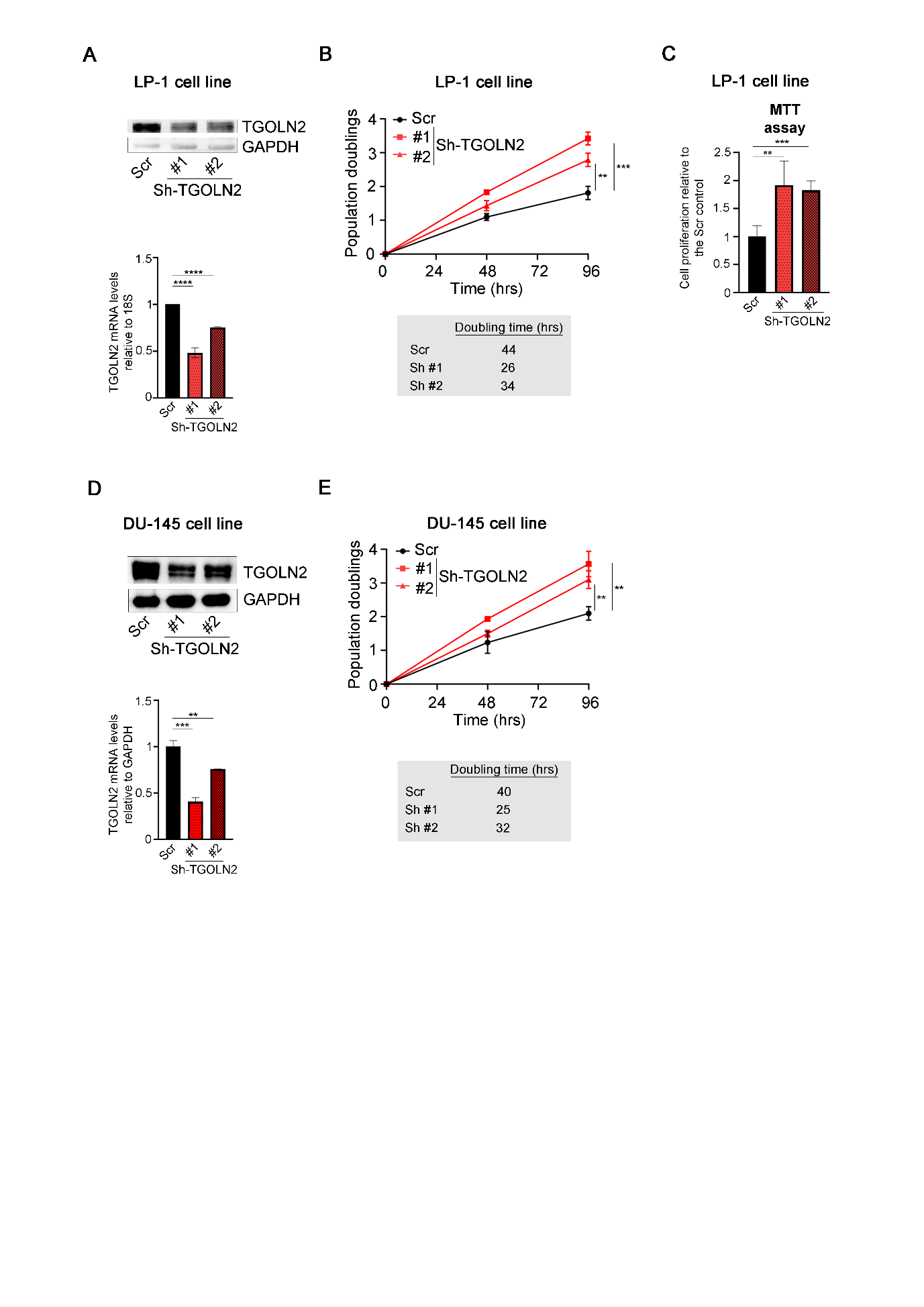
**

**Supplementary Figure S6. TGOLN2 has tumour suppressor-like properties in *in vitro* models of MM and PrC.**

A) TGOLN2 levels in the LP-1 MM cell line with TGOLN2 downmodulating constructs.

Top) Western blot showing the levels of the TGOLN2 protein in the LP-1 MM cell line expressing either a control plasmid (Scr) or Sh-RNA constructs targeting TGOLN2 (sh-TGOLN2 #1 and #2). GAPDH was used as loading control. Bottom) RT-qPCR showing mRNA levels of TGOLN2 in the same cell lines.

B) Proliferation assay in LP-1 MM cells with TGOLN2 downmodulation. Same cell lines as in A) were used to perform a proliferation assays (top) and assess doubling times (bottom).

C) MTT assay of LP-1 MM cell lines with TGOLN2 downmodulation. Same cells as in A) were used to assess metabolic activity through MTT assays.

D, E) Same as (A, B) except that experiments were performed in the DU-145 PrC cell line

Histograms represent the mean +/- SD of three independent experiments.

**
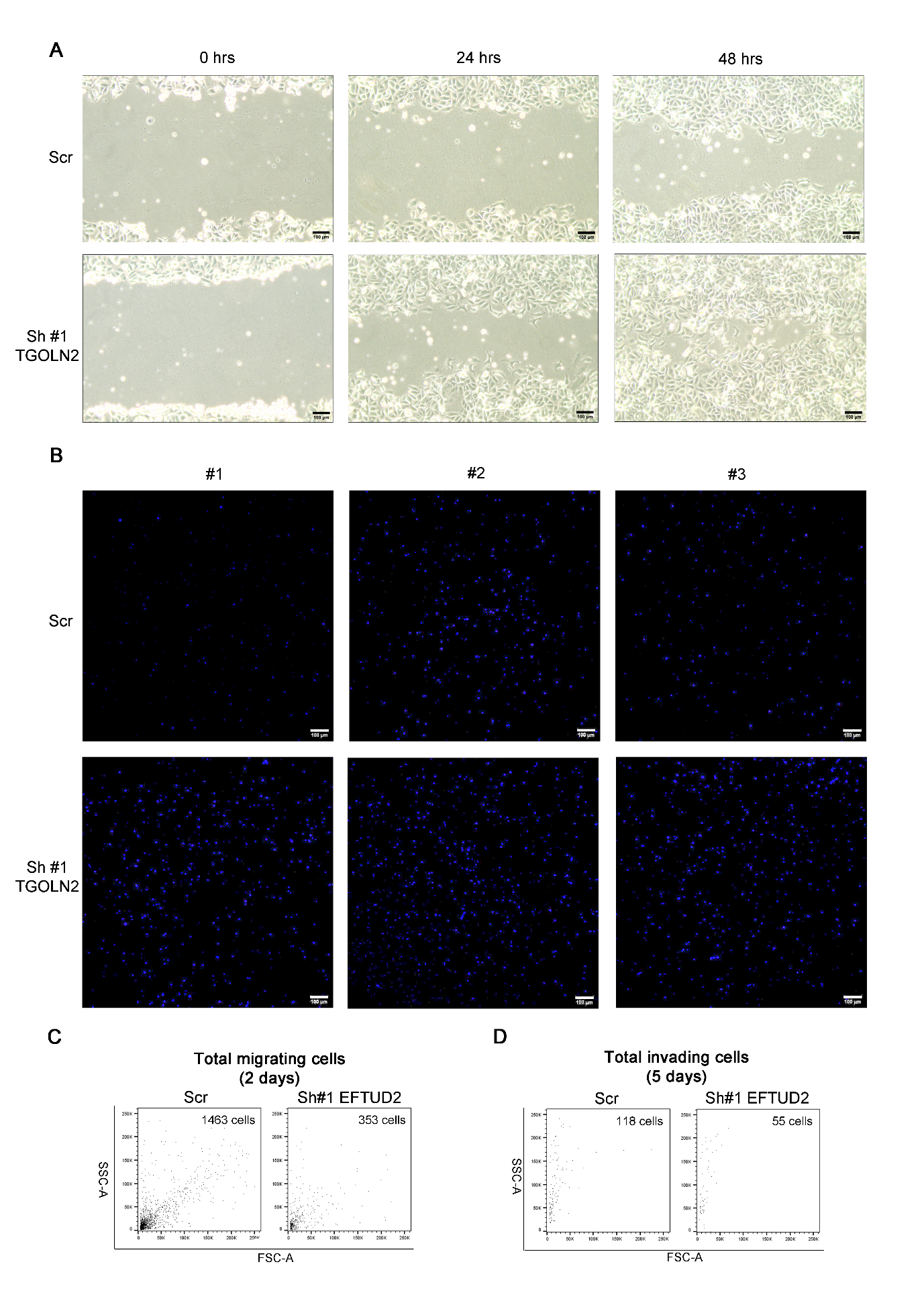
**

**Supplementary Figure S7. TGOLN2 and EFTUD2 affect migration and invasion of RPMI MM and MCF-7 BC cell lines, respectively.**

A-B) Representative images of wound healing (A) and invasion (B) assays performed in MCF-7 BC cells. Same cells as in Fig.5A were used to perform both scratch and invasion assays.

A) Once the scratch (wound) was performed on adherent plated cells using a 200μl pipette tip (0hrs), cells were monitored after 24 and 48 hrs for restoration of wound gaps. Error bars: 100μm.

B) Cells capable to digest the matrigel-coated transwells and “invade” the lower side of the membrane. Error bars: 100μm.

C-D) Cytofluorimetric counting of invading and migrating RPMI MM cells.

Same cells as in Fig.6A were used to perform both migration (C) and invasion (D) assays using transwell chambers. C) Total number of cells migrating through a representative transwell chamber. D) Total number of cells invading through a representative matrigel-coated chamber. Quantitations are representative of three independent experiments.
