# Supplementary material for: CancerHubs: a systematic data mining and elaboration approach for identifying novel cancer-related protein interaction hubs
Source: Brief Bioinform. 2024 Dec 7;26(1):bbae635. doi: 10.1093/bib/bbae635 (PMC11631132; doi:10.1093/bib/bbae635)
Supplement: Supplementary_Materials_and_methods_ok_bribio_bbae635 [file supplementary_materials_and_methods_ok_bribio_bbae635.docx]

**Comparative analysis**

Comparative analysis was performed by intersecting the lists of genes ranked by *network score* generated using *CancerHubs* with those generated by CancerGeneNet [1] or stored in the NCG database [2]. Specifically, for each tumour considered (MM, BC, PrC, CRC and PaC), we extracted the list of merged *seed* and *interactor* genes using *CancerHubs*. After ranking genes based on *network scores*, we defined as “outliers” those genes with *network scores* significantly higher than the mean, i.e., > mean + 2xSD, and considered them as putatively cancer-related. Lists of *CancerHubs* outlier genes for each tumour were intersected with either the list of cancer-related genes obtained through the “CancerBrowser” feature of CancerGeneNet (complexity level 3, all types of interactions, assessed on July 11, 2024) or the merged list of canonical and putative cancer-driver genes listed in NCG (assessed on July 11, 2024) for that specific tumour. We then calculated, for each tumour, the percentage of shared genes between the outlier gene list of *CancerHubs* and the list generated by either CancerGeneNet or NCG.

**GSEA**

GSEA was performed using the GSEA software [3,4]. For each cancer considered (MM, BC, PrC, CRC and PaC) we used the full merged list of genes (*seeds* and *interactors*) ranked by *network scores* and the H (Hallmark) gene sets. Gene sets were considered significantly enriched when both Nominal *p-values* and FDR *q-values* were below 0.05.

**Datamining of *network scores***

Having generated ranked-gene lists for each cancer considered, we implemented a datamining approach to define common-cancer or cancer-specific protein *hubs*.

We started from the lists of ranked *seed* genes and considered only the top-50 *network scoring* genes of each cancer. By intersecting the lists together, we defined: 1) common-cancer genes and 2) cancer-specific genes.

Among the top-50 scoring *seed hubs* of each cancer, none was shared among all five tumours. Hence, subsequent analyses were performed on the top-12 genes present in at least 3 out of the 5 cancers analysed and such genes were defined as “common-cancer *seed* hubs”.

Among the top-50 scoring *seed hubs* of each cancer, several genes were instead found to be present exclusively in one specific cancer. Hence, for subsequent analyses, we focused our attention on the top-20 genes specific of each cancer and such genes were defined as “cancer-specific *seed* hubs”.

The same approach was employed on the lists of ranked *interactor* protein hubs.

Among the top-50 scoring *interactor hubs* of each cancer, only two were shared among all five tumours. Hence, subsequent analyses were performed on the top-13 genes present in at least 4 out of the 5 cancers analysed. Such genes were defined “common-cancer *interactor* hubs”.

Among the top-50 scoring *interactor hubs* of each cancer, only few genes were found to be present exclusively in one specific cancer. Hence, for subsequent analyses, we focused our attention on the top-6 genes specific of each cancer. If the number was lower than 6, analyses were performed on less than 6 genes. Such genes were defined “cancer-specific *seed* hubs”.

For common-cancer gene analyses, we also combined *seed* and *interactor* hub lists.

We either 1) intersected the top-50 scoring *seed* and *interactor* hubs lists of each cancer with one another and analysed only common genes or 2) merged the top-12 common cancer *seed* hubs with the top-13 common *interactor* hubs and analysed the merged list.

**GO term analysis**

GO term analyses on common-cancer or cancer-specific genes were performed using the Generic Gene Ontology (GO) term finder online tool (https://go.princeton.edu/cgi-bin/GOTermFinder assessed on July 3, 2023) as described in [5].

Analyses were performed on the top-12 common-cancer *seed* or top-13 common-cancer *interactor* hubs and on the top-20 cancer-specific *seed* or top-6 cancer-specific *interactor* hubs.

Analyses were also performed on the top-12 common-cancer *seeds* + top-13 common-cancer *interactors* merged list and on the 21 gene hubs present indistinctively either as *seeds* or *interactors* among the top-50 *network scoring* genes of every cancer considered. In the latter case GO terms were summarized using ReviGO [6].

**Cell culturing**

All the cell lines used in this study were obtained from the ATCC.

HEK-293T virus packaging, MCF-7 BC, DU-145 PrC and LP-1 and RPMI-8226 MM cells were cultured and maintained at 37°C with 5% CO_2_ in a thermal incubator. HEK-293T and LP-1 cells were grown in IMDM, MCF-7 in DMEM/F-12 and DU-145 and RPMI-8226 in RPMI-8226 1640 media. All media were supplemented with 100 U/mL penicillin, 100 μg/mL streptomycin, 1% glutamine and 10% FBS, with the exception of IMDM for LP-1 cells which was supplemented with 20% FBS. Cells were tested monthly for mycoplasma contamination.

**Lentiviral particle production**

To produce lentiviral particles HEK-293T packaging cells were transiently transfected using calcium phosphate precipitation with envelope plasmid VSV-G, packaging plasmids PMDLg/pRRE and pREV, and transfer vectors of interest as described in [7]. Specifically, we used the following: MISSION pLKO.1-puro Non-Target shRNA Control (Sigma-Aldrich, cat. no. SHC016), MISSION pLKO.1-puro sh-TGOLN2#1 (Sigma-Aldrich, cat. no SHCLNG, clone TRCN0000312400), MISSION pLKO.1-puro sh-TGOLN2#2 (Sigma-Aldrich, cat. no SHCLNG, clone TRCN0000159517), GIPZ Lentiviral shRNA control vector (Horizon Discovery), GIPZ Lentiviral Human EFTUD2 shRNA#1 (Horizon Discovery, clone V2LHS_28167), GIPZ Lentiviral Human EFTUD2 shRNA#2 (Horizon Discovery, clone V3LHS_639417).

**Transduction of MCF-7, LP-1, DU-145 and RPMI-8226 cells**

One million MCF-7, LP-1, DU-145 or RPMI-8226 cells were transduced with 1,5 mL of crude viral supernatants expressing either the MISSION pLKO.1-puro Non-Target shRNA Control, sh-TGOLN2#1 or shTGOLN2#2 vectors or the GIPZ Lentiviral shRNA control vector, sh-EFTUD2#1 or sh-EFTUD2#2 vectors, in the presence of 4 μg/mL Polybrene. After centrifugation at 500 × g for 45 min at room temperature, the cells were incubated at 37°C in 5% CO2 for 12- 15 h. After media switch, cells were cultured for another 24-48 hrs and subsequently selected for 4 days with puromycin (final concentration 2μg/ml). At the 4^th^ day of puromycin selection TGOLN2 and EFTUD2 levels were assessed by western blotting and RT-QPCR.

**Western blotting**

SDS-PAGE, followed by western blotting, was performed on protein extracts derived from (i) MCF-7 BC cells transduced with lentiviruses expressing either the pLKO.1 control vector or shRNAs targeting TGOLN2 or (ii) RPMI-8226 MM cells transduced with lentiviruses expressing either the GIPZ control vector or shRNAs targeting EFTUD2. Briefly, cells were collected and lysed in radioimmunoprecipitation assay buffer (10 mM Tris-HCl [pH 7.4], 1% sodium deoxycholate, 1% Triton X-100, 0.1% SDS, 150 mM NaCl, and 1 mM EDTA [pH 8.0]) as described in [8].

TGOLN2 levels were determined using α-TGOLN2 antibodies (1:1.000 dilution; Sigma Life Science, cat. no. HPA012723), while EFTUD2 levels were determined using α-EFTUD2 antibodies (1:1.000 dilution; Invitrogen, cat. no. PA5-96559). β-Actin and GAPDH were used as loading controls and were detected using α-β-Actin (1:4.000 dilution; Sigma-Aldrich, cat. no. A5441) and α-GAPDH (1:1.000 dilution; Cell Signaling, cat. no. 2118) antibodies, respectively.

Western blot signals were detected using the Super Signal West Pico Plus chemiluminescent substrate (Thermo Fisher Scientific, cat. no. 34577), according to the manufacturer’s instructions, and an iBright imaging system (Thermo Fisher Scientific).

**mRNA extraction and RT-qPCR**

Total RNA from MCF-7, LP-1, DU-145 and RPMI-8226 cells was extracted using TRIzol reagent (Thermo Fisher Scientific, catalog no. 15596026) and the RNeasy Mini Kit (Qiagen, catalog no. 74104) as previously described [9]. Briefly, 1μg of total RNA was then reverse transcribed using random primers and the SuperScript III First-Strand Synthesis SuperMix (Invitrogen, catalog no. 11752-050). RT-qPCR on neo-synthesized cDNA was then performed using either the GoTaq qPCR Master Mix (Promega, catalog no. A6001) or TaqMan Universal PCR Master Mix (Thermo Fisher Scientific, catalog no. 4304437) on a StepOnePlus system (Thermo Fisher Scientific).

The following primers or TaqMan probes were used:

-EFTUD2 FWD: 5’-CAATATCATGGACACTCCAGGAC-3’

-EFTUD2 REV: 5’-CGGTCAATCTTGTTGATGCACA-3’

-GAPDH FWD: 5’-ATGACCCCTTCATTGACC-3’;

-GAPDH REV: 5’-GAAGATGGTGATGGGATTTC-3’;

-TGOLN2 Taqman probe: Hs00197728_m1 (ThermoFisher Scientific cat. No. 4331182)

-18S Taqman probe: Applied Biosystems, catalog no. 4333760F

**Proliferation assay and doubling time calculation**

Population doubling calculation was performed in MCF-7 BC, LP-1 MM, DU-145 PrC and RPMI-8226 MM cells expressing control vectors or constructs for downmodulation of either TGOLN2 or EFTUD2. Viable cells were counted by Trypan Blue exclusion at 0, 48 and 96hrs of cell culturing and population doubling times were calculated at the 48 and 96hrs time points.

**MTT assay**

Metabolic activity of MCF-7 BC and LP-1 and RMPI-8226 MM cells expressing control vectors or constructs for downmodulation of either TGOLN2 or EFTUD2 was assessed through MTT assays using the MTT Assay Kit from Abcam (Abcam, cat. no. 211091) following the manufacturer’s instructions. Briefly, 20.000 cells/well were seeded in 96-well plates and incubated in complete medium for 48 hrs. After 48 hrs the media was replaced with serum-free media in the presence of the MTT reagent. After a subsequent 3 hr incubation at 37°C, 5% CO2, the MTT solvent was added and cells were shaked on an orbital shaker for 15 minutes. Absorbance at 590 nm was then read on a microplate reader (iMARK, BIORAD)

**Migration assay**

Cell migration was assessed by either wound healing assays, in MCF-7 BC adherent cells, or by transwell migration assays, in RPMI-8226 MM suspension cells.

For wound healing assays, MCF-7 control and sh-TGOLN2#1 cells were grown in 6-well plate culture plates. After reaching confluency, a thin "wound" was introduced to the confluent cells by scratching with a 200μl pipette tip.

Cells’ wound healing capacity was then monitored at 24 and 48hrs post-wound generation through image capturing using a Leica brightfield microscope with a C-Mount 0.35X camera. Wound gap areas were quantified using ImageJ.

Migration capacity of RPMI-8226 MM suspension cells was instead assessed performing transwell assays. Briefly, prior to subculturing to migration chambers, cells were grown to 70/80% confluency in media containing 10% FBS. 5x10^4^ cells were then washed and resuspended in 0.2 ml of serum-free culture media and added to the top inserts of 24-well migration chambers (Corning Costar 6.5 mm transwells, cat. no. 3422) with 0.5 ml of media containing 10% FBS as chemoattractants added to the bottom of the wells.

Cell invasion chambers were incubated for 2 days in a humidified incubator at 37°C, 5% CO_2_ and cell migration was assessed by counting the number of cells migrating to the bottom of the well through flow cytometry using a BD FACS CANTO II flow cytometer and the BD FACSDIVA and FlowJo softwares.

**Invasion assay**

Invasion capacity of both MCF-7 and RPMI-8226 MM cells was assessed using matrigel-coated transwells. Experiments were performed as described in the “Migration assay” paragraph except that the membrane inserts were previously coated with matrigel and that chambers were incubated at 37°C, 5% CO_2_ for 3 days (MCF-7 cells) or 5 days (RPMI-8226 cells).

Cell invasion was assessed by quantifying the number of cells reaching the bottom well using either 1) a spinning disk CREST-X-Light-V2 (CrestOptics) mounted on a fully automated Nikon Ti inverted microscope, after addition to the cells of one drop of NucRed Live 647 Reagent (Thermo Fisher; Cat. No: R37106) (MCF-7 cells) or 2) flow cytometry employing a BD FACS CANTO II flow cytometer and the BD FACSDIVA and FlowJo softwares (RPMI-8226 cells).

**Statistics**

Statistical *P*-values were calculated using unpaired *t*-tests. ns: *P* > 0.05; **: *P* < 0.01; ***: *P* < 0.001; ****: *P* < 0.0001. All histograms shown represent the mean +/- SD or SEM of a minimum of three independent experiments.

**References**

1. Iannuccelli M, Micarelli E, Surdo PL, et al. CancerGeneNet: linking driver genes to cancer hallmarks. Nucleic Acids Research 2020; 48:D416–D421

2. Dressler L, Bortolomeazzi M, Keddar MR, et al. Comparative assessment of genes driving cancer and somatic evolution in non-cancer tissues: an update of the Network of Cancer Genes (NCG) resource. Genome Biol 2022; 23:35

3. Mootha VK, Lindgren CM, Eriksson K-F, et al. PGC-1α-responsive genes involved in oxidative phosphorylation are coordinately downregulated in human diabetes. Nat Genet 2003; 34:267–273

4. Subramanian A, Tamayo P, Mootha VK, et al. Gene set enrichment analysis: A knowledge-based approach for interpreting genome-wide expression profiles. Proc. Natl. Acad. Sci. U.S.A. 2005; 102:15545–15550

5. Mancino M, Lai G, De Grossi F, et al. FAM46C Is an Interferon-Stimulated Gene That Inhibits Lentiviral Particle Production by Modulating Autophagy. Microbiol Spectr 2023; 11:e05211-22

6. Supek F, Bošnjak M, Škunca N, et al. REVIGO Summarizes and Visualizes Long Lists of Gene Ontology Terms. PLoS ONE 2011; 6:e21800

7. Miluzio A, Cuomo A, Cordiglieri C, et al. Mapping of functional SARS-CoV-2 receptors in human lungs establishes differences in variant binding and SLC1A5 as a viral entry modulator of hACE2. eBioMedicine 2023; 87:104390

8. Manfrini N, Mancino M, Miluzio A, et al. FAM46C and FNDC3A Are Multiple Myeloma Tumor Suppressors That Act in Concert to Impair Clearing of Protein Aggregates and Autophagy. Cancer Res 2020; 80:4693–4706

9. Manfrini N, Ricciardi S, Miluzio A, et al. High levels of eukaryotic Initiation Factor 6 (eIF6) are required for immune system homeostasis and for steering the glycolytic flux of TCR-stimulated CD4+ T cells in both mice and humans. Developmental & Comparative Immunology 2017; 77:69–76
